# Immunogenicity and Efficacy of a Novel Multi-Antigenic Peptide Vaccine Based on Cross-Reactivity between Feline and Human Immunodeficiency Viruses

**DOI:** 10.3390/v11020136

**Published:** 2019-02-03

**Authors:** Bikash Sahay, Alek M. Aranyos, Meerambika Mishra, Andrew C. McAvoy, Marcus M. Martin, Riuyu Pu, Sayaka Shiomitsu, Keijiro Shiomitsu, Michael J. Dark, Missa P. Sanou, Shannon R. Roff, Mobeen H. Rathore, Janet K. Yamamoto

**Affiliations:** 1 Department of Infectious Diseases and Immunology, College of Veterinary Medicine, University of Florida, P.O. Box 110880, Gainesville, FL 32611-0880, USA; sahayb@ufl.edu (B.S.); aaranyos1@ufl.edu (A.M.A.); meerambikamishra@ufl.edu (M.M.); acmcavoy145@gmail.com (A.C.M.); pur@ufl.edu (R.P.); sshiomitsu@ufl.edu (S.S.); 2 Biovalion: Clinical Research, 7951 Ponds Edge Ln, Zephyrhills, FL 33540-1973, USA; marcus.m.martin@gmail.com; 3 Department of Small Animal Clinical Sciences, College of Veterinary Medicine, University of Florida, P.O. Box 100116, Gainesville, FL 32610, USA; kshiomitsu@ufl.edu; 4 Department of Comparative, Diagnostic & Population Medicine, College of Veterinary Medicine, University of Florida, P.O. Box 100123, Gainesville, FL 32610-0123, USA; darkmich@ufl.edu; 5 Merck & Co., 770 Sumneytown Pike, North Wales, PA 19486, USA; missa.sanou@merck.com; 6 Charles River Laboratories Inc., 15 Worman’s Mill Court, Suite I, Frederick, MD 21701, USA; Shannon.Roff@crl.com; 7 Education, and Service (UF CARES), University of Florida Center for HIV/AIDS Research, Jacksonville, FL 32209-6810, USA; Mobeen.Rathore@jax.ufl.edu

**Keywords:** FIV vaccine, HIV-1, T cell epitopes, cytotoxic T lymphocyte, polyfunctional T cells

## Abstract

For the development of an effective HIV-1 vaccine, evolutionarily conserved epitopes between feline and human immunodeficiency viruses (FIV and HIV-1) were determined by analyzing overlapping peptides from retroviral genomes that induced both anti-FIV/HIV T cell-immunity in the peripheral blood mononuclear cells from the FIV-vaccinated cats and the HIV-infected humans. The conserved T-cell epitopes on p24 and reverse transcriptase were selected based on their robust FIV/HIV-specific CD8^+^ cytotoxic T lymphocyte (CTL), CD4^+^ CTL, and polyfunctional T-cell activities. Four such evolutionarily conserved epitopes were formulated into four multiple antigen peptides (MAPs), mixed with an adjuvant, to be tested as FIV vaccine in cats. The immunogenicity and protective efficacy were evaluated against a pathogenic FIV. More MAP/peptide-specific CD4^+^ than CD8^+^ T-cell responses were initially observed. By post-third vaccination, half of the MAP/peptide-specific CD8^+^ T-cell responses were higher or equivalent to those of CD4^+^ T-cell responses. Upon challenge, 15/19 (78.9%) vaccinated cats were protected, whereas 6/16 (37.5%) control cats remained uninfected, resulting in a protection rate of 66.3% preventable fraction (*p* = 0.0180). Thus, the selection method used to identify the protective FIV peptides should be useful in identifying protective HIV-1 peptides needed for a highly protective HIV-1 vaccine in humans.

## 1. Introduction

The development of an effective HIV-1 vaccine for prophylaxis and immunotherapy is essential for controlling the spread and contributing to the eradication of global HIV-1 infection [1,2,3,4]. The most promising phase-III HIV-1 (RV144) vaccine trial conferred protection rate of 31.2% in the overall risk group (consisting of three sub-groups), but afforded only 3.7% in the high-risk sub-group [5,6]. The goal of this prime-boost vaccine approach was to induce both antibody and cellular immunity against HIV-1 for prophylaxis [5,6,7]. In comparison, higher rates of protection were observed in animal AIDS models with simian and feline immunodeficiency virus (SIV and FIV) vaccines [8,9,10]. The immunization of rhesus macaques with experimental CMV-vectored SIV *gag/pol/env* vaccine resulted in a transient infection with complete clearance of infection in 50% of the vaccinated macaques, but with persistent infection in the remaining animals [8,11]. The clearance of SIV infection was attributed to anti-SIV CD8^+^ T-cell immunity generated by the vaccine. A recent field study in Western Australia with a commercial inactivated FIV vaccine conferred protection of 56% preventable fraction in client-owned, outdoor-access cats after receiving annual boosts for ≥3 years [10]. The commercial FIV vaccine, consisting of inactivated dual subtype-A and -D FIV strains, induced both anti-FIV neutralizing antibodies (NAbs) and T-cell immunity [9,12,13,14]. The efficacy of the vaccine-induced NAbs was limited to FIV viruses closely related in the envelope (Env) sequence, such as those existing in FIV subtypes A and D [9,13]. In contrast, the T-cell immunity induced by the commercial and its prototype FIV vaccines conferred protection against distinctly heterologous FIV strains from the same (homologous) and heterologous subtypes, indicating that anti-FIV T-cell immunity may have a broader breadth of immunity than NAb immunity induced by the commercial vaccine [12,13]. Therefore, both animal AIDS models demonstrate the key role of anti-lentiviral T-cell immunity in vaccine prophylaxis. 

The importance of anti-FIV T-cell immunity in conferring prophylaxis by the commercial and prototype FIV vaccines further raises a question on whether a T cell-based FIV vaccine can confer substantial protection against FIV. Current studies were undertaken to address this query and a few additional critical questions, such as: (1) What are the protective T-cell activities against AIDS lentiviruses? (2) What are the best approaches in selecting protective T-cell epitopes that do not mutate? (3) Are there deleterious epitopes on AIDS lentiviruses, such as HIV-1 and FIV, which should be deleted from the vaccine immunogen? (4) Which vaccine design would best augment the antiviral activities of a T cell-based lentivirus vaccine? Moreover, the FIV infection of domestic cats causes feline AIDS, a significant health concern for veterinary medicine [15]. Consequently, ongoing studies also provide novel insights in developing an effective second-generation FIV vaccine. However, our overarching goal of the current studies is to utilize the FIV vaccine model of HIV/AIDS to address above queries, which should aid in the development of a highly effective HIV-1 vaccine for humans.

## 2. Materials and Methods

### 2.1. The Immune Analyses Used in Selecting Protective T-Cell Epitopes on FIV

The protective FIV peptide epitopes were selected by their potential to induce high levels of T-cell proliferation and cytokine production as the polyfunctional T-cell activities for both FIV-vaccinated cats and HIV^+^ human subjects. The CD4^+^ and CD8^+^ T-cell proliferation were determined by the flow cytometry (fluorescence-activated cell sorter [FACS])-based carboxyfluorescein diacetate succinimide ester (CFSE) proliferation analyses with a positive threshold of ≥0.5% CFSE^low^ as previously described [16]. IFNγ and IL-2 production were measured by IFNγ and IL-2 ELISpot analyses using feline or human IFNγ and IL-2 ELISpot module kits from R&D Systems (Minneapolis, MN) using manufacturer’s protocol [16]. The positive threshold for ELISpot analyses was ≥50 spot forming units (SFU)/10^6^ peripheral blood mononuclear cells (PBMC). The production of perforin, granzyme A (GrzA), GrzB, and CD107a in the human CD4^+^ and CD8^+^ T cells represented the HIV/FIV lentiviral-specific CD4^+^ and CD8^+^ CTL activities of HIV^+^ subjects. The levels of perforin, GrzA, and GrzB present in the CD4^+^ and CD8^+^ T cells were assessed by FACS-based intracellular cytolysin/cytotoxin staining (ICS), and the CD107a on cell surface determined by multicolor FACS with a positive threshold for FACS-based ICS at 0.1% fluorescence as previously described [17,18]. Human blood samples were obtained from the University of California at San Francisco (UCSF) and the University of Florida Center for HIV/AIDS Research, Education and Service (UF CARES) in Jacksonville using the protocols approved by the Institutional Review Board at UF as previously described [17,18].

### 2.2. The Approaches for Selecting Mutation-Resistant, Protective FIV Epitopes 

The FIV protein with the most conservation between FIV and HIV sequences were determined to be polymerase-reverse transcriptase (Pol-RT) followed by Pol-integrase and Gag-p24 [19]. As a result, the T-cell proliferation and the IFNγ and IL-2 productions to the overlapping peptide pools from HIV and FIV p24 and RT proteins were assessed using the T cells and PBMC from prototype FIV-vaccinated cats and HIV^+^ human subjects as previously described [17,18]. Each peptide pool consisted of three-to-four overlapping FIV or HIV p24 peptides and four-to-five overlapping peptides of FIV or HIV RT, both with an overlap of 6–10 amino acids. RS Synthesis LLC (Louisville, KY, USA) synthesized the overlapping peptides spanning the full length of p24 and RT (described in [17,18]), the individual protective FIV peptides, the overlapping protective peptides (Fp14-3/Fp14-4, FRT3-3/FRT3-4, FRT7-1/FRT7-2), and the counterpart HIV peptides. The selection of the protective FIV peptide pools and the individual protective FIV peptides in the selected peptide pools are described in the Result 3.1. Upon selection of individual protective FIV peptides, these peptides and some of the multiple antigenic peptides MAPs (MAP2v, MAP5) were tested for their ability to directly enhance or suppress the in vitro FIV infection. This FIV enhancement/suppression assay is similar to the in vitro HIV enhancement/suppression assay previously reported [18] except for the following modifications. The T-cell mitogen (ConA)-stimulated feline PBMC from multiple uninfected specific pathogen free (SPF) cats were used instead of human PBMC from multiple HIV-negative subjects, varying dilutions of FIV instead of HIV, and the focus on both FIV and counterpart HIV peptides for the analysis instead of only HIV peptides. Each peptide or MAP was assayed 2–4 times, each time using PBMC from a different cat.

### 2.3. In Sillico HLA Algorithms

HLA algorithms used are Net-MHC 4.0 server for HLA-A, HLA-B, and HLA-C [20,21], Net-MHCII 2.3 server for HLA-DRB1 [22,23], and NetCTL 1.2 server for potential HLA supertype of the potential CD8^+^ CTL epitope [24,25]. Net-MHC 4.0 and Net-MHCII 2.3 are ranked by binding affinity [20,21,22,23], whereas NetCTL 1.2 are scored by a combination of MHC-I binding affinity, sensitivity, and specificity [24,25].

### 2.4. Constructing the MAP Vaccine Immunogens into A Vaccine

The MAP vaccine consisted of four MAPs of protective FIV peptide epitopes mixed in Fort Dodge-1 (FD-1) adjuvant (Fort Dodge Animal Health, Fort Dodge) identical to the adjuvant used for the commercial FIV vaccine [12,14,26]. Each MAP contained four identical FIV peptides on the amino-end of the lysine backbone and palmitic acid (i.e., palmitate; C16) or Tat sequence (GRKKRRQRRRPPQQ) on the carboxyl-end of the lysine backbone. Palmitate is a toll-like receptor (TLR) ligand, which allows the MAP to enter the host cells by binding for example to the TLR on the cell surface [27,28]. Whereas, HIV-1 Tat sequence allows the conjugated molecule to enter the cells using the transduction domain (GRKKRRQRRR) of the Tat [29]. The rationale for using MAP vaccine design is to deliver multiple copies of mutation-resistant protective FIV epitopes into the antigen presenting cells (APCs) which in turn can present these epitopes using major histocompatibility complex (MHC) I and II to the CD8^+^ and CD4^+^ T cells, respectively, to induce their effector function against FIV [12,27,28]. LifeTein LLC (South Plainfield, NJ, USA) synthesized the MAP4 immunogen with the protective peptide Fp9-3 and the MAPs with overlapping protective peptides Fp14-3/Fp14-4 (MAP3), FRT3-3/FRT3-4 (MAP2/2v), and FRT7-1/FRT7-2 (MAP5).

### 2.5. Animals

Specific pathogen free (SPF) cats were purchased from Marshal BioResources/Liberty Research, Inc. (Waverly, NY, USA) or bred by the Laboratory of Comparative Retrovirology and Immunology at the University of Florida. These cats were SPF for the reported feline viral infections [30]. All breeding and animal procedures were performed according to the policy and protocols (#201701838 and #201702539) approved by the Institutional Animal Care and Use Committee (IACUC) at the University of Florida.

### 2.6. MAP Vaccines and Vaccination

The SPF cats in Group 1a received concurrent subcutaneous (SC) and intradermal (ID) immunizations with either 400 µg total of MAP2/3/4t or 500 µg total of MAP2/3/5 mixture. Each of the MAP mixtures consisted of three MAPs (MAP2, MAP3, and MAP4t or MAP5) in a total volume of 1.5 mL of FD-1 adjuvant supplemented with feline IL-12 (4 µg). MAP2/3/4t vaccine consisted of 150 µg MAP2, 150 µg MAP3 and 100 µg MAP4t, whereas MAP2/2v/3/5 vaccine contained 140 µg MAP2, 140 µg MAP2v, 110 µg MAP3, and 110 µg MAP5. MAP2 and MAP2v contain overlapping peptide FRT3-3/FRT3-4 with either one aa (methionine) deletion or lysine substitution of methionine, respectively as described in Table 1. Group 1a received MAP2/3/4t vaccine for the first SC/ID vaccination and MAP2/2v/3/5 vaccine for the last two SC/ID vaccinations at 1:2 vaccine volume consisting of 0.5 mL of SC vaccination together with 1.0 mL of ID vaccination. The SPF cats in Groups 1b and 1c were immunized with MAP2v/3/4/5 vaccine containing four MAPs (MAP2v, MAP3, MAP4 and MAP5). An equal amount of 100 µg per MAP (400 µg total MAPs) was mixed in a total volume of 1.5 mL FD-1 adjuvant alone for SC vaccination and 1.0 mL of FD-1 adjuvant supplemented with 4 µg of feline IL-12 (R&D Systems, Minneapolis, MN) for ID vaccination.

All MAPs (MAP2, MAP3, MAP4, MAP5), except for MAP4t, were synthesized with palmitic acid (C16) on carboxyl-end. Only MAP4t was synthesized with HIV-1 Tat instead of palmitate on the carboxyl-end. Due to high in vitro toxicity of Tat-conjugated MAP4t, the MAP4t in the vaccine was replaced with MAP5 which had a low in vitro toxicity much like MAP2, MAP3, and MAP4. (All assays used palmitated MAPs.) All vaccines were freshly mixed before each vaccination according to the recommendation of the manufacturer of FD-1 adjuvant. All MAP vaccinations, including the control immunizations were administered at six-week interval. 

The four SPF control groups consisted of three cats (Group 2a) with SC/ID adjuvant immunization, five cats (Group 2b) with SC/ID PBS immunization, three cats each (Group 2c) with SC or ID adjuvant immunization, and two cats (Group 2d) without any immunization. All control groups except for Group 2d received three immunizations.

### 2.7. Monitoring Vaccine Immunogenicity

Immune analyses were performed at 3-6 weeks post-second vaccination depending on the vaccine groups, and three and six weeks post-third vaccination for all vaccine groups. Immune analyses consisted of CD4^+^ and CD8^+^ T-cell proliferation using FACS-based CFSE-proliferation with a threshold of 0.5% CFSE^low^ as previously described [16,17,18], and IL-2 and IFNγ ELISpot analyses for production of these cytokines with a positive threshold of 50 SFU/10^6^ PBMC as previously described [16]. In addition, at six weeks post-third vaccination before the challenge, additional blood samples were collected for PBMC to perform FIV peptide- or MAP-specific perforin, GrzA, GrzB, IL-2, and IFNγ mRNA production analyses. A million PBMC from eight MAP vaccinated cats (four each from Groups 1b and 1c) and six control cats (five from Group 2c, one from Group 2b) were stimulated with 3 µg/mL of MAP or FIV peptides, or 0.2 µg/mL T-cell mitogen, *Staphylococcus* enterotoxin A (SEA) for 14 hours at 37°C. The culture media consisted of AIMV media supplemented with 5% heat-inactivated fetal bovine serum and 25 µg/mL gentamycin. After incubation, plates were spun down to collect the cells for total RNA isolation using Aurum Total RNA isolation Kit (Bio-Rad Laboratories, Hercules, CA). The complementary DNA (cDNA) generated using iScript RT Kit (Bio-Rad Laboratories) were amplified with a set of primers and TaqMan probe (Table S8) using Mic qPCR machine (Bio Molecular Systems, Australia). The mean CT values for all the calculations used glyceraldehyde 3-phosphate dehydrogenase (GAPDH) as an internal normalization control. Transcript levels for stimulated groups were presented as a fold change over their media controls and adjuvant control groups. A higher than the two-fold difference to control is considered significant when bars are above the dotted line (Figure S3).

### 2.8. FIV Challenge and Monitoring FIV Infection 

All vaccinated and control cats were challenged with 1.25 CID_50_ of subtype-B FIV_FC1_ at six weeks post-third vaccination for the initial challenge via intravenous injection under sedation. Challenged cats were monitored for FIV infection by virus isolation [9,12], FIV proviral PCR [9,12,33], reverse transcriptase (RT) assay [34,35], and by detection of FIV antibodies using immunoblot analysis [9]. All FIV-positive control cells or sera used FIV_FC1_, while negative control were from SPF cats. The MAP vaccination did not interfere with FIV immunoblot analysis. The protected/vaccinated cats and the uninfected control cats received a high-dose of 25 CID_50_ at the final challenge to determine the strength and duration of the vaccine protection. Virus load assay is a modification of the one described [36]. PBMC from FIV-challenged cats (10, 1 × 10^2^, 1 × 10^3^, 1 × 10^4^, 1 × 10^5^, 1 × 10^6^) were co-cultured for three weeks with T-cell enriched PBMC (7.5 × 10^5^/well) from SPF cats in a total volume of 1.0 mL per well in duplicate cultures per cell dilution using 48-well culture plates. T-cell enrichment was performed by stimulating with concanavalin A (T-cell mitogen) for three days in HuIL-2 containing RPMI culture media (RPMI 1640 supplemented with heat-inactivated 10% fetal bovine serum, 10 mM HEPES, 50 µL/mL gentamycin, and 40 U/mL HuIL-2) and reculturing for additional 3–6 days with culture media. Culture supernatants were harvested and fresh culture media added every 3–4 days (twice a week). Virus production was determined by measuring the RT activity in the collected culture supernatants, whereas upon termination of cultures, the cells were tested for proviral PCR [33]. Results on FIV load in PBMC are presented with the cell dilution (log^−X^ or 10^−X^) of infected cells needed to detect FIV. The highest cell dilution 10^-6^ titer as six and the lowest detectable titer of 0.5 based on duplicate cultures.

### 2.9. Statistical Analyses and Protection Rate

The statistical comparison between each vaccine group and the combined control Group 2a–d, was determined by two-tailed Fisher’s exact test. Similarly, a statistical comparison was made between combined control Group 2a–d and the combined vaccine Groups 1abc or the combined vaccine Groups 1bc. The statistical comparison of the in vitro results was based on unpaired two-tailed Welch’s *t*-test with unequal variance. The protection rate was based on percent preventable fraction, which factors in the infection rate of the control group in defining the protection rate. Percent preventable fraction is equal to the incidence of the infected control group minus the incidence of infected vaccine group divided by the incidence of the infected control group [13].

## 3. Results

### 3.1. Selection of Mutation-Resistant, Protective T-cell Epitopes on FIV

**Figure 1:**
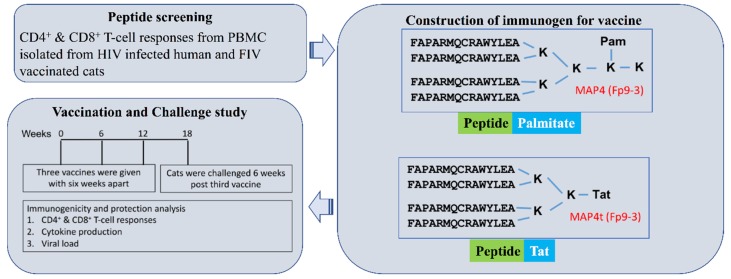
Schematic of the study: Evolutionarily or lentivirally conserved peptides were selected based upon their ability to induce CD8^+^ T-cell proliferation in isolated peripheral blood mononuclear cells (PBMC) of infected human HIV^+^ patients and feline immunodeficiency virus (FIV) vaccinated cats. These selected peptides (shown in Table 1) were tagged at their carboxyl-end with palmitate or Tat peptide sequence for their sufficient intracellular delivery for enhancement of CD8^+^ T-cell responses. These peptides in multiple antigen peptides (MAP) formulation with Fort Dodge-1 (FD-1 adjuvant were tested for immunogenicity and protection in cats using FIV_FC1_ as the challenging virus. The efficacy of the vaccine was monitored by viral load, CD4^+^ and CD8^+^ T-cell responses, and cytokine production by the cells. Note that the MAP4t was used only in the first vaccination of vaccine Group 1a.

To determine the mutation-resistant protective T-cell epitopes, the study was designed in a three-step process. First, small overlapping peptides were generated from FIV or HIV genome and used as a testing tool for CD8^+^ T-cell responses in vitro. The peptides that showed a significant increase in CD8^+^ T-cell responses both in feline and human infected PBMC were selected for generating immunogens for an animal study, as described previously [17,18]. The four peptides of the same kind were tagged with palmitic acid or Tat peptide using three–five lysine residues [16] (Figure 1) to enhance intracellular permeability of these peptides for efficient antigen presentation and loading on MHCI molecule. The 8–12-week-old SPF cats were used to test the immunogenicity and protection as depicted in Figure 1. The protective FIV peptide epitopes were selected by their potential to induce CD4^+^ and CD8^+^ T-cell proliferation, IFNγ and/or IL-2 production, and in some cases production of perforin, GrzA, GrzB, and CD107a to represent CTL activity (see Methods 2.1). HIV^+^ human responders of the CD4^+^ and CD8^+^ T-cell proliferation and IFNγ production were observed substantially more to the FIV RT peptide pools than to the FIV p24 peptide pools (Table S1). These results support the notion that sequence conservation exists between FIV and HIV RT more than those between FIV and HIV p24 as previously reported [4,17,18]. Since non-specific activation of CD4^+^ T cells can enhance FIV and HIV-1 infection [4,37], the selection of conserved protective peptide pools was based predominantly on CD8^+^ T-cell responses and to a lesser degree on CD4^+^ CTL activity. The Fp9 and Fp14 pools of FIV p24 and the FRT3 and FRT7 pools of FIV RT induced the most CD8^+^ T-cell proliferation, as well as the most CD4^+^ and CD8^+^ CTL activities in the T cells from HIV^+^ individuals (Table S1). These observations suggest that these FIV peptide pools are conserved with the counterpart HIV peptide pools on HIV p24 and RT [17,18]. Remarkably, Fp9, Fp14, and FRT7 pools also induced CD8^+^ T-cell proliferation in ≥50% of the FIV-vaccinated cat responders, whereas the FRT3 pool induced CD8^+^ T-cell proliferation in 36% of the cat responders (Table S1). The individual peptides in these peptide pools were next evaluated along with the peptides in the Fp3, Fp4, and Fp10 peptide pools (Table S2). The latter three p24 pools induced IFNγ production in a reasonable percentage (10%) of HIV^+^ responders (Table S1). However, the selection of additional peptides was not pursued with RT peptide pools based on the findings from HIV studies indicating more HIV-1 p24 peptides than RT peptides induce HIV-specific CTL activities [4,38,39,40,41]. 

**Figure 2:**
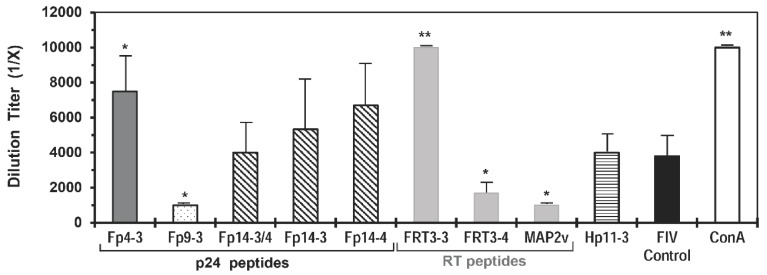
**Direct effect of FIV peptides and MAP2v on in vitro FIV infection.** The FIV peptides and MAP tested are FIV reverse transcriptase peptides FRT3-3, FRT3-4, and MAP2v (grey bar); FIV p24 peptides Fp9-3 (dotted bar); Fp14-3, Fp14-4, and overlapping Fp14-3/Fp14-4 (Fp14-3/4) (slanted line bar); and p24 peptide Fp4-3 (dark grey bar) as the positive control for peptide-induced enhancement. MAP2v contains the overlapping FRT3-3/FRT3-4 peptide. T-cell mitogen control (concanavalin A, ConA) (white bar) is used as a strong positive control for enhancement, whereas the HIV p24 peptide Hp11-3 (horizontal line bar) is included as a negative control. A peptide or MAP is considered to be enhancing or suppressing FIV infection when the average result post stimulation with the peptide or MAP is significantly higher or lower, respectively than the average result of the FIV virus control (black bar). Significant differences exists between the average result of the peptide- or MAP-stimulated triplicate cultures and those of the virus control cultures when the bar has (*) for *p* < 0.05 or (**) for *p* < 0.01. Although all peptides with corresponding MAPs were performed three-six times in smaller studies, the figure shows a typical result from one of the two studies with all of the peptides shown performed together. Each study is performed using PBMC from a different specific pathogen free (SPF) cat. MAP5 containing overlapping Fp10-2/Fp10-3 has been tested, but not shown.

According to the selection based on cross-reactivity to HIV, the peptides Fp9-3 of Fp9 pool, Fp14-4 of Fp14 pool, FRT3-3 of FRT3 pool, and FRT7-1 of FRT7 pool induced the highest percentage of HIV^+^ responders with CD8^+^ T-cell proliferation among the individual peptides in the designated pool (Table S2). Additionally, the CD4^+^ CTL frequency to peptides Fp9-3, Fp14-4, and FRT3-3 was the highest among the peptides in their respective pool. The peptide immediately adjacent to Fp14-4 and FRT7-1 are the Fp14-3 with the second highest human CD8^+^ T-cell proliferation and the FRT7-2 with the highest CD8^+^ CTL frequency, respectively. The FRT3-4 peptide overlapping FRT3-3 at the carboxyl-end was selected over peptide FRT3-2 based on the FRT3-4 peptide inducing substantially higher CD8^+^ and CD4^+^ CTL frequencies than FRT3-2 peptide. The Fp9-3 peptide was selected as a single peptide since it possessed far greater cross-reactivity to CD8^+^ T-cells, CD8^+^ CTLs, and CD4^+^ CTLs of HIV^+^ individuals than its overlapping peptide on the amino-end. The peptides in Fp10 pool were not selected for the current vaccine trial due to the low cross-reactivity to HIV. Thus, a single peptide and three sets of two overlapping peptides were selected as the mutation-resistant, protective FIV p24 and RT peptides (Table 2). All selected FIV peptides either suppressed (Fp9-3, FRT3-4, FRT7-1/FRT7-2) or had no effect (Fp14-3, Fp14-4) on the in vitro FIV infection of primary PBMC, except for the enhancement of in vitro FIV infection induced by FRT3-3 peptide (Figure 2, summary in Table 2). However, the MAP2v with the overlapping peptide FRT3-3/FRT3-4 suppressed FIV infection of the PBMC. These results from the in vitro studies suggest that the epitopes on viral RT and p24 must be selected carefully by excluding the enhancing epitopes of FIV infection (e.g., Fp4-3) or by overlapping the enhancing epitope with suppressive epitopes (e.g., FRT3-3 with suppressive FRT3-4). 

Since these FIV peptides induced T-cell responses with the PBMC from HIV^+^ human subjects, these peptides should be able to bind to the human leukocyte antigens (HLA) to mediate these T-cell responses. According to the in silico HLA analyses, all seven of these FIV peptides possessed the potential to bind to HLA class I (HLA-A, HLA-B, and/or HLA-C) and six of them also possessed the potential to bind to HLA class II (HLA-DRB1) (Table 2, last two columns).

### 3.2. Designing the Vaccine Immunogens

The Fp9-3 peptide of MAP4 contains a protective epitope [18] with high sequence conservation with all four FIV subtypes A, B, C, and D analyzed (Table 1, column 2), but only marginal sequence conservation with its counterpart HIV peptide (Table 1, column 5). In the case of the highly-conserved protective peptides Fp14-4, FRT3-3, and FRT7-1, each of these FIV peptides and its adjacent peptide (Fp14-3, FRT3-4, FRT7-2) with high protective activities were synthesized into a single overlapping peptide (Table 1, column 4) containing potentially two protective epitopes. All six peptides possessed a sequence identity to one-to-three FIV subtypes (Table 1, column 2). Since FIV isolates are so far distributed into five subtypes with subtypes A and B most prevalent worldwide [31], the combined coverage of these peptides should be broad enough to cover the majority of FIV isolates prevalent worldwide.

### 3.3. Immunogenicity of the MAP Vaccines

**Figure 3:**
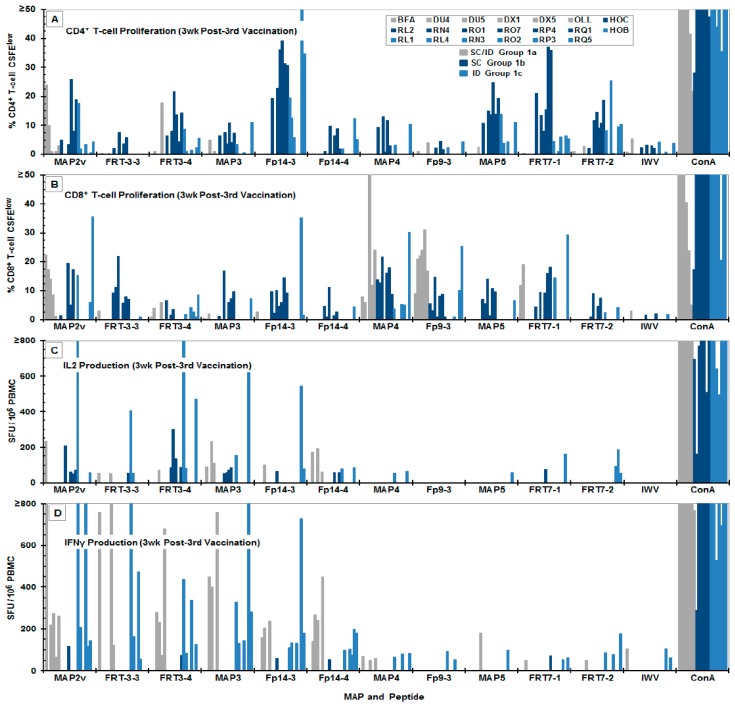
FIV MAP/peptide-specific T-cell proliferation and cytokine production of the PBMC from vaccinated cats at three weeks post-third vaccination. The results from CD4^+^ (**A**) and CD8^+^ (**B**) T-cell proliferation and the IL-2 (**C**) and IFNγ (**D**) production of the PBMC from the MAP-vaccinated cats are shown in response to the in vitro stimulation with MAP or FIV peptide. The vaccinated cats in Group 1a received concurrent subcutaneous plus intradermal (SC/ID) vaccinations with triple-MAP vaccine (grey bars), while those in Groups 1b and 1c received either subcutaneous (SC) (dark blue bars) or intradermal (ID) (blue bars) vaccinations with quadruplicate-MAP vaccine, respectively. The identification codes of each vaccinated cat are shown in the top right inset of panel A. Each bar represents the average response from a single vaccinated cat after subtraction of the average value of the results from eight control cats, with the exception of the mitogen control. Only bars at ≥0.5% CFSE^low^ threshold for CD4^+^ and CD8^+^ T-cell proliferation or at ≥50 SFU/10^6^ PBMC threshold for IL-2 and IFNγ production are shown.

Most the FIV peptides and MAPs induced low-titer of CD4^+^ and CD8^+^ T-cell proliferation and low-titer of IFNγ and IL-2 production at post-second vaccination (Figure S1) in cats. However, the number of responders varied depending on the peptide and MAP used for the in vitro stimulation. The IL-2 and IFNγ production to MAP4 and its peptide Fp9-3 had only one responder and two-to-three responders, respectively (Figure S1C,D). The IL-2 and IFNγ production to MAP4 and Fp9-3 continued to be of low magnitude with few-to-no responders at both three and six weeks post-third vaccination (Figure 3C,D and Figure S2C,D). In comparison to the cytokine responses, low-to-high magnitudes of CD4^+^ and CD8^+^ T-cell proliferation responses to MAP4 and peptide Fp9-3 was detected post-second vaccination (Figure S1A,B). A substantially more CD8^+^ T-cell proliferation than the CD4^+^ T-cell proliferation to MAP4 and Fp9-3 were observed at three weeks post-third vaccination (Figure 3A,B). By six weeks post-third vaccination, the CD4^+^ T-cell proliferation more than CD8^+^ T-cell proli-feration to Fp9-3 diminished, whereas the CD8^+^ T-cell proliferation to MAP4 maintained while CD4^+^ T-cell proliferation to MAP4 increased (Figure S2A,B). 

**Figure 4:**
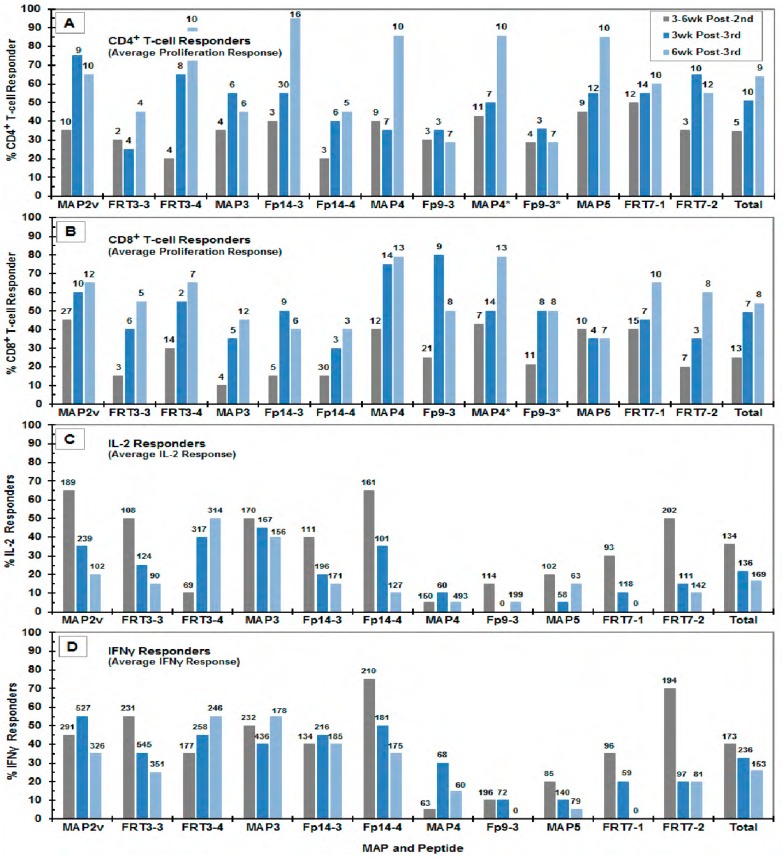
Average percent responder rates and average magnitudes of CD4^+^ and CD8^+^ T-cell proliferation and the cytokine production at the three time points tested. The average percent responder rates of the CD4^+^ (**A**) and CD8^+^ (**B**) T-cell proliferation and the IL-2 (**C**) and IFNγ (**D**) production of the PBMC from the MAP-vaccinated cats are shown in response to in vitro stimulation with MAP or FIV peptide. The three time points tested are 3-6 weeks post-second vaccination (grey bars), three weeks post-third vaccination (dark blue bars), and three weeks post-third vaccination (blue bars), as shown in the insert of panel A. The average magnitude of response are shown above each bar. The three bars in the last column above “Total” represents the total or overall average of each time point with the overall average magnitude above each bar. Note that the CD4^+^ and CD8^+^ T-cell responder rates to MAP4 and Fp9-3 at six weeks post-third vaccination is missing the results from Group 1a.

A more consistent increase in the average magnitude and the number of CD8^+^ T-cell proliferation to all peptides and MAPs was observed starting at three weeks post-third vaccination (Figure 2B) which continued to increase at six weeks post-third vaccination, with the exception of Fp9-3, Fp14-3, and MAP5 (Figure S2B and Figure 4B). In contrast, the elevated average numbers of CD4^+^ T-cell responders to Fp9-3, FRT7-2, MAP2v, and MAP3 at three weeks post-third vaccination decreased slightly, but were compensated by a concurrent increase in average magnitude by six weeks post-third vaccination, with an exception of those to MAP3 with no change in magnitude (Figure 4A).

Contrary to the T-cell proliferation, the number of IL-2 and IFNγ responders had a decreasing trend with exceptions of IL-2 responses to FRT3-4, Fp9-3, MAP4, and MAP5, and the IFNγ responses to FRT3-4, Fp14-3, and MAP3 (Figure S1C,D, Figure 3C,D, Figure S2C,D; summarized Figure 4C,D). Sixty-four percent (7/11 MAP/peptide stimulants) of the IFNγ responses increased in magnitude between post-second vaccination and three weeks post-third vaccination, but by six weeks post-third vaccination the magnitude of all IFNγ responses decreased (Figure 4D). In contrast, the average magnitude of the IL-2 responses had an increase in 45% (5/11) of the peptide/MAP stimulations between post-second vaccination and six weeks post-third vaccination and 45% (5/11 stimulants) between three and six weeks post-third vaccinations (Figure 4C). The decreasing number of IL-2 and IFNγ responders and the increasing trend of the responders to CD4^+^ and CD8^+^ T-cell proliferation are also depicted in the average of the total responses (Figure 4, above “Total”). 

At six weeks post-third vaccination, FIV peptide/MAP-specific IL-2 and IFNγ mRNA levels were evaluated along with mRNA levels to CTL-associated GrzA, GrzB, perforin, and CD107a. In general, the cats in Group 1c more than those in Group 1b responded with higher IL-2 and IFNγ mRNAs to FIV peptides and MAPs (Figure S3). The GrzA and CD107a mRNA levels in Group 1b than in Group 1c were either significantly or substantially higher to MAP5 and FRT7-1/2 mixture (a mixture of single peptides FRT7-1 and FRT7-2, plus overlapping FRT7-1/FRT7-2). This observation suggested that FRT7-1/2 mixture induced the CTL-associated activities in cats from Group 1b more than Group 1c. In comparison, a single cat each from Group 1c responded at high GrzB levels to the majority of MAPs and peptide mixtures except to MAP4 and MAP5. Furthermore, only Group 1c had substantially elevated levels of perforin mRNA to FRT3-3/4 mixture.

### 3.4. MAP Vaccine Efficacy against Challenge with Pathogenic Subtype B FIV (FIV_FC1_)

The SPF cats vaccinated thrice with the MAP vaccine composed of three to four MAPs depending on the vaccine group (Table 3 and Table S3). The vaccine Group 1a receiving three MAPs conferred the lowest protection rate of 20% preventable fraction, whereas Groups 1b and 1c vaccinated with all four MAPs afforded a protection rate of 100% and 77.1%, respectively (Table 3). Significant protection rates were observed when the combined Control Group 2 (2a–d) was compared to the vaccine Group 1b (*p* = 0.0152), Groups 1bc (*p* = 0.0057), or combined Groups 1abc (*p* = 0.0180). Overall, the efficacy results from the current vaccine trial demonstrate that the T cell-based FIV MAP vaccine containing at least four protective MAPs can confer significant protection against FIV challenge.

The four unprotected/vaccinated cats showed no sign of enhancement of FIV infection (Table S3). Additionally, one (cat OLL) showed a delay in infection by three weeks and had a lower FIV load in all tissues tested compared to the other unprotected/vaccinated cats or the infected control cats with only a few exceptions (R05 in three tissues, HOI in all tissues). The average viral load in the PBMC, bone marrow (BM) cells, thymus (Th) cells, and mesenteric lymph node (mLN) cells of the four unprotected/vaccinated cats at 24 wpc (the time-point of termination) were only slightly lower than those of the infected control cats except for the average load in the thymus (Table S3, bold values). The average FIV load in the tissues found highest in the BM followed by thymus, PBMC, and mLN cells for both unprotected/vaccinated and infected-control groups. The FIV loads in the BM cells of the infected-control group were significantly higher than the FIV loads in the PBMC (*p* = 0.009), thymus cells (*p* = 0.05), and mLN (*p* = 0.0011) cells. Remarkably, even the virus load in the BM cells from unprotected/vaccinated cats were significantly higher than those in the mLN cells (*p* = 0.0309). Nonetheless, the FIV loads in the PBMC and the mLN cells did not differ significantly in both unprotected/vaccinated (*p* = 0.3665) and infected-control (*p* = 0.2395) groups. 

The 13 protected/vaccinated and five challenged/uninfected control cats were monitored for infection over an additional 18 weeks before challenging them with a final high-dose of FIV. Thus, at 48 weeks post-third vaccination, these cats were re-challenged with a moderately high dose (25 CID_50_) of FIV_FC1_ that infected all the cats.

### 3.5. Statistical Comparison between the Unprotected/Vaccinated and the Protected/Vaccinated Cats

Four vaccinated cats remained unprotected after the low dose challenge; three of them belonged to vaccine Group 1a and one unprotected cat was from vaccine Group 1c. The vaccinated cats that acquired protection distributed among different groups as indicated. Three of the protected cats were from Group 1a; whereas all seven cats from Group 1b and six cats from Group 1c (note Group 1bc with 13 protected cats) remain protected. The average responder rates of the unprotected/vaccinated cats were consistently lower in both CD4^+^ and CD8^+^ T-cell proliferation when compared to the protected cats from Group 1bc and combined Group 1 (or 1abc) (Table 4). The statistical difference in CD4^+^ T-cell responder rate was observed at post-second vaccination (*p* = 0.0078) and at three weeks post-third vaccination (*p* = 00003) when compared to the protected cats in Group 1bc; however, there was no difference by six weeks post-third vaccination. When compared to all protected cats from Group 1, significant differences were observed by three (*p* = 0.0038) and six (*p* = 0.0125) weeks post-third vaccination with also a trend observed at post-second vaccination (*p* = 0.0732). A significant difference was also observed in the CD8^+^ T-cell responder rates at both post-second vaccinations (*p* = 0.0335) and three weeks post-third vaccination (*p* = 0.0004) when unprotected cats were compared to protected cats in Group 1, and at three weeks post-third vaccination (*p* = 0.0018) when compared to protected cats in Group 1bc, as well as a trend at post-second vaccination (*p* = 0.0648). 

The difference between the two comparisons (unprotected group *versus* protected cats from Group 1bc or Group 1) is attributed to the deliberate omission of the results to MAP4 and Fp9-3 peptides from six vaccinated cats from Group 1a in Table 4, Table 5, Table S5, and Table S6, resulting from insufficient recovery of PBMC from these cats at six weeks post-third vaccination. As a result, the MAP4/Fp9-3-specific CD4/CD8 T-cell results from the unprotected group at six weeks post-third vaccination was entirely omitted from the analysis in Table S5. The MAP4/Fp9-3-specific CD4/CD8 T-cell results at six weeks post-third vaccination for the individual cats in Table 5 were included in the analysis for cats in Group 1bc, but was unavailable in the analysis for the six cats in Group 1a. The summarized average results in Table 4 are also affected since the average results from Table 5 and Table S5 are used.

All unprotected/vaccinated cats did not produce IL-2 in response to Fp9-3, FRT7-1, MAP4, and MAP5 at all the time-points tested (Table S5). Even the protected cats from the vaccine Group 1bc responded feebly for the IL-2 production with Fp9-3, FRT7-1, and MAP4 at all time points except for FRT7-1 at post-second vaccination (Table S6). Among the critical observation of the study was the responder rates and the magnitudes of MAP/peptide-specific IFNγ production substantially higher than those of IL-2 production in the unprotected cats at the all-time point tested, with a significant difference at three weeks post-third vaccination (responder: *p* = 0.0061; magnitude: *p* = 0.0245) (Table S5). In contrast, both the responder rates and the average magnitudes of MAP/peptide-specific IL-2 and IFNγ production in the protected cats from vaccine Group 1bc remained about the same at all time-points (Table S6). Fp14-3, FRT7-1, and MAP5 induced higher CD4^+^ T-cell proliferation than CD8^+^ T-cell proliferation throughout all time-points tested (Table S6). Conversely, Fp9-3, FRT3-3, and MAP4 induced higher CD8^+^ T-cell proliferation than CD4^+^ T-cell proliferation consistently at three and six weeks post-third vaccination. Remarkably, Fp9-3 and MAP4 induced generally the most CD8^+^ T-cell proliferation with low-to-moderate CD4^+^ T-cell proliferation and often with the least number of IFNγ responder at all time-points.

## 4. Discussion

According to the World Health Organization, estimated 1.8 million new cases have been reported in 2017, although the anti-retroviral therapy (ART) has been used to control the spread of the infection [4,42]. The ART therapy is successful in managing the virus and increasing the lifespan of the infected individual. However, it is unable to cure the infection; additionally, the recurring cost of the treatment warrants a vaccine against the virus [43,44]. Most of the HIV researchers used SIV as their surrogate for HIV-1 infection and vaccine trials; here, we have been using FIV as a model for HIV vaccine as a cost-effective research tool. In the past few decades, neutralizing antibodies were the target; however, recognition of non-neutralizing antibodies and antibody-dependent enhancement of infection forced us to find alternatives [4,7,45,46,47]. To avoid such infection-enhancing vaccines, the current study took an approach to select epitopes (i) devoid of enhancing epitope from the target proteins, (ii) select epitopes from the target proteins that are known to induce CD8^+^ CTL responses, (iii) select epitopes for enhanced polyfunctional CD8^+^ T-cell responses, and (iv) screen for epitope that produces least amount of pro-inflammatory cytokines [4,48].

Antigen presentation is a complex process and essential for the protection conferred by the vaccine. To screen the target peptides, current study relied on recall responses from the HIV^+^ human PBMC and vaccinated feline PBMC. As mentioned earlier, the epitopes were screened for CD8^+^ T-cell proliferation and against pro-inflammatory cytokine production. The rationale behind the screening against the cytokine production was to avoid epitopes that could release cytokines that could lead to (i) increase in viral infection and (ii) cause more immunopathology. The selected peptides were palmitate or tagged with Tat sequence to increase cell-permeability for an efficient CD8^+^ T-cell responses (Figure 1). 

### 4.1. The Selection of Potential Protective HIV Peptides

The protective FIV peptides were considered conserved when they induced antiviral T-cell activities by the T cells from both prototype FIV-vaccinated cats and HIV^+^ individuals. This observation suggested that these conserved protective peptides may also induce protective T-cell activities against HIV. The counterpart HIV peptides to Fp9-3, Fp14-3/Fp14-4, FRT3-3/3-4, and FRT3-4 are Hp10-3, Hp15-3, HRT3-3, and HRT3-4, respectively, which also induced HIV-specific CD4^+^ CTL, CD8^+^ CTL, and polyfunctional T-cell activities when tested with isolated T cells and PBMC from HIV^+^ subjects (Table S4) [17,18]. Much like the FIV counterpart peptides, these HIV peptides also possessed a high potential to bind to HLA class I based on in silico MHC-I algorithms, as well as Los Alamos National Laboratory (LANL) database for functional HIV specific CTL activity [20,24,49]. However, the HIV HRT7 peptide pool and its feline counterpart FRT7, displayed only a modest CD4^+^ and CD8^+^ T-cell proliferation responses (Table S4) as described in our earlier study [17]. 

In the previous study, the 27% responder rate of CD8^+^ T-cell proliferation to HRT11 pool was the highest among all 21 HIV RT peptide pool, and even to its counterpart FRT11 pool had a responder rate of 25% [17]. In current preliminary studies with T cells from HIV^+^ human subjects, the peptide HRT11-4 had the most CD8^+^ T-cell proliferation with broad binding potential to the HLA class-I supertypes (B8, C3, C6, A1/A3, A2). In addition, it induced the most CD8^+^ CTL responder rate and response frequency among the five peptides in the HRT11 pool (Table S4). The high CD8^+^ CTL responder rate may be associated with the three CTL epitopes that have strong-to-moderate specificities for the five HLA supertypes (strong: A3, B8, B39; moderate: B62, A2). The CD8^+^ T-cell proliferation to its counterpart FIV peptide FRT11-5 was not detected due to the low number (0/3) of subjects tested; however, substantial CD8^+^ CTL responder rate (82%) and response frequency (0.5152 or 51.5%) were observed. In support of the latter finding as determined by NetCTL 1.2 algorithm, the FRT11-5 sequence had strong specificities and score for three HLA supertypes (B62, B58, A3) of which two supertypes (A3, B62) were the same as those identified for HRT11-4. In summary, the HIV-1 peptides Hp10-2, Hp15-3/4, HRT3-3/HRT3-4, and HRT11-4 (the counterparts to Fp9-3, Fp14-3/Fp14-4, FRT3-3/FRT3-4, and FRT11-5) may be outstanding candidates as the anti-HIV T-cell epitopes for HIV vaccine and immunotherapy. Overall, conservation in both function (Table 2) and sequence (Table 1) was observed between the FIV peptides and their counterpart HIV peptides.

Perhaps a noteworthy observation is that CD4^+^ T-cell proliferation using selected protective FIV peptides were generally lower in the CD4^+^ T cells from HIV^+^ subjects than those from FIV-vaccinated cats, even though the FIV peptide-specific CD8^+^ T-cell proliferation of the vaccinated cats was comparable to the CD8^+^ T-cell expansion of the HIV^+^ subjects (Table 1 and Table S2). This observation is most likely due to the CD4^+^ T-cell population being affected by HIV infection causing CD4^+^ T-cell depletion and/or suppression of CD4^+^ T-cell activity as previously observed [50,51]. Furthermore, the antiretroviral drugs could have also affected the CD4^+^ T-cell responses since the majority of the new HIV^+^ subjects were on antiretroviral therapy (ART) [52,53]. This observation also suggests that these HIV^+^ subjects still retained the CD8^+^ T-activities, indicating that the counterpart HIV peptides may be useful even for immunotherapy in HIV^+^ subjects on ART. The therapeutic use of these protective FIV peptides in FIV^+^ cats may be the first step in evaluating this possibility. In support of such study, therapeutic vaccination of HIV^+^ subjects with modified vaccinia Ankara vectored-HIV*gag/pol/nef/gp120* vaccine induced CD8^+^ T-cell responses to the conserved regions of HIV that reduced peak viremia and delayed viral rebound upon treatment interruption [3,54,55].

### 4.2. The in vitro Selection of Protective FIV Peptides Compared to the Peptide-Specific Immunity of Vaccinated Cats

Since the protective FIV peptides screening was based upon an in vitro method, there was a possibility that the selected peptide may respond differently in cats. The average responder rate of the CD4^+^ and CD8^+^ T-cell proliferation to the seven peptides from the selection studies were similar to the results from three weeks post-third vaccination of the protected MAP-vaccinated cats (Table 1 and Table S7). The average results were 55.1% CD4^+^ T-cell responder and 57.1% CD8^+^ T-cell responder in the selection study (Table S7), whereas the average results were 64.8% peptide-specific CD4^+^ T-cell responders and 54.9% peptide-specific CD8^+^ T-cell responders in the protected cats from Group 1bc in the current trial at three weeks post-third vaccination (Table S7). A slight difference in the average number of responders was anticipated since the blood samples for the selection study were collected at 3-4 weeks post-last vaccination from the prototype FIV-vaccinated cats with at least four vaccinations. The average responder rate of the proliferation responses between the CD4^+^ and the CD8^+^ T cells did not significantly differ in both selection study (Table 1 and Table S7, *p* = 0.8768) and the current MAP vaccine trial (Table S7, *p* = 0.3195). More importantly, no statistical difference observed in the CD4^+^ T-cell proliferation responses to the FIV peptides between the selection study and the vaccine trial at three (*p* = 0.4491) and six (*p* = 0.1446) weeks post-third vaccination, as well as at post-second vaccination (*p* = 0.5780). Similarly, no statistical difference was observed in the CD8^+^ T-cell proliferation responses to the same peptides between the selection study and the vaccine trial at three (*p* = 0.8293) and six (*p* = 0.8300) weeks post-third vaccinations, but not at post-second vaccination (*p* = 0.0700). Notably, the CD8^+^ T-cell proliferation responses were significantly lower at post-second vaccination than at three (*p* = 0.0003) and six (*p* < 0.0001) weeks post-third vaccination. These results were taken together further indicate that CD8^+^ T-cell responses to these peptides significantly increases after the third vaccination, and that the T-cell epitope selection approach concurs with the T-cell immunity results from the MAP-vaccinated cats.

The average number of responders of the CD4^+^ T-cell proliferation was slightly higher than that of CD8^+^ T-cell proliferation throughout all time-points in the vaccine trial (Tables S6 and S7), whereas almost identical responder rates were detected between these CD4^+^ and CD8^+^ T-cell subpopulations in the peptide selection study (Table 2 and Table S7). Although our goal was to select for FIV peptide with more CD8^+^ T-cell responses, the FIV peptide selection based on conservation with HIV resulted in selecting FIV peptides that induced almost equivalent levels of CD4^+^ and CD8^+^ T-cell proliferation in the T cells from the prototype FIV-vaccinated cats. Remarkably, these conserved FIV peptides induced more CD8^+^ T-cell proliferation than CD4^+^ T-cell proliferation with the T cells from HIV^+^ human subjects (Table 2, *p* = 0.0129). Thus, FIV peptides Fp10-2 and Fp10-3 that has more CD8^+^ than CD4^+^ T-cell proliferative ability in the prototype FIV-vaccinated cats (Table S2) and in our preliminary immunogenicity study (unpublished observation) should be considered as the additional peptides to include in a vaccine even though their conservation to HIV are generally lower than those tested in the current vaccine trial. However, these peptides are highly conserved among FIV subtypes A, B, and C (data not shown).

### 4.3. MAP Vaccine Immunity and Efficacy

Overall, robust CD4^+^ and CD8^+^ T-cell proliferation responses to one or more peptides and MAPs were observed in each MAP-vaccinated cat, which peaked by six weeks post-third vaccination (Figure S1A,B, Figure 3A,B, Figure S2A,B). In contrast, the initial robust IL-2 and IFNγ responses to multiple peptides and MAPs generally decreased by six weeks post-third vaccination (Figure S1C,D, Figure 3C,D, Figure S2C,D, Figure 4C,D). 

The current study indicates that the MAP vaccine containing four MAPs (Groups 1b and 1c) confers more protection than those containing triple MAPs (Group 1a). Besides requiring all four MAPs in each vaccination for most protection, this difference in protection rates can be attributed to the difference in the vaccination routes used. Group 1a received a combined SC and ID vaccination, whereas Groups 1b and 1c received a single route of either SC or ID vaccination, respectively. The low challenge dose of 1.25 CID_50_ used in the current study is more similar to those of natural transmission [13]. The inability to protect against the high dose (25 CID_50_) at 48 weeks post-vaccination in the current trial suggests that a higher and/or more frequent administration of MAP immunogen, or including additional MAP with peptides, such as Fp10-2/Fp10-3, may be required to achieve long-lasting protection of higher multitude. 

### 4.4. Correlates of MAP Vaccine Protection

A significant correlation of protection with elevated CD4^+^ T-cell proliferation was observed in the protected/vaccinated cats more than in the unprotected/vaccinated cats (Table 4). The fact that the protected cat RL4 from Group 1c had only a strong CD4^+^ T-cell proliferation without detectable CD8^+^ T cell proliferation and IL-2/IFNγ production further supported the importance of CD4^+^ T-cell activities, as well as suggesting the importance of the quality of FIV MAP/peptide-specific CD4^+^ T-cell activities (Table 5). Notably, more protected/vaccinated cats responded with MAP/peptide-specific IL-2 production and at higher magnitudes than unprotected/vaccinated cats, whereas more unprotected cats responded with MAP/peptide-specific IFNγ production than protected cats (Table 4). Thus, IL-2 production associates with protection, while IFNγ production associates with no protection. 

The importance of CD8^+^ T-cell responses may also exist since significantly higher responder rate was observed in the protected/vaccinated cats than in the unprotected/vaccinated cats from both Group 1bc (*p* = 0.0018) and the total combined Group 1 (*p* = 0.0004) at three weeks post-third vaccination (Table 4). Nevertheless, it is somewhat remarkable that a protein/peptide vaccine can maintain the CD4^+^ and CD8^+^ T-cell proliferation up to six weeks post-third vaccination. Both Fp9-3 and MAP4 induced the most CD8^+^ T-cell proliferation and frequently with the least number of IFNγ responder at all time-points tested (Table S6). In fact, 4/7 protected cats (HOC, RL2, RO7, RP4) from SC-vaccinated Group 1b possessed substantially more CD8^+^ than CD4^+^ T-cell proliferation (Table 5). In comparison, only 1/6 protected cat (RQ5) from ID-vaccinated Group 1c and 1/4 unprotected cat (OLL) from SC/ID vaccinated Group 1a had a higher CD8^+^ than CD4^+^ T-cell proliferation. This observation further supports the argument that ID vaccination may not induce as much CD8^+^ T-cell immunity in cats even though the vaccine immunogen levels were identical between Groups 1b and 1c. Besides Group 1a having more immunogen delivered by ID route than by SC route, this group received the least amount of CD8^+^ T-cell-inducing Fp9-3 peptide immunogen by receiving only single MAP4t vaccination. Overall, both CD4^+^ and CD8^+^ T-cell immunity generated by MAP vaccine may be important for conferring protection. 

### 4.5. Future Directions in Improving T Cell-Based FIV/HIV Vaccines

Current results suggest the need for improvement in the vaccine immunogen to confer significant protection against a high challenge dose and protection for at least a year. Since the current MAP vaccine formulation provided significant CD4^+^ T-cell responses, the vaccine could be improved by including FIV peptides (e.g., Fp10-2/Fp10-3) that induce more anti-FIV CD8^+^ T-cell activities. A simple approach is to increase the MAP vaccine dose, while a more complex approach is to express these conserved protective peptides using an expression vector as a vaccine delivery system known to enhance CD8^+^ T-cell activity [56]. In order to maximize the expression of the target peptides, multiple gene sequences of these peptides must be linked. The linking of the peptide gene sequences must be carefully designed since new epitopes could arise during the bridging of the targeted peptides that could enhance infection by neutralizing or decreasing the efficacy of the protective T-cell epitopes. Another important issue is the vector itself should target antigen presenting cells, such as macrophages and dendritic cells, but should not express its structural gene since non-specific stimulation can enhance FIV and HIV infection [4,57]. A more comprehensive approach is to include linear NAb epitope(s) in combination with these protective T-cell epitopes. As of to date based on PubMed search of publications, a linear NAb epitope for FIV is still unknown except for the FIV surface envelope (Env) that neutralizes FIV only when CrFK cells are used [58,59]. More importantly, linear NAb epitopes on HIV Env-transmembrane (Env-TM) have been reported [4,60]. Thus, the combination of the linear NAb epitopes of HIV TM together with the conserved protective T-cell epitopes on HIV Gag and Pol should lead to a highly effective HIV vaccine for humans. 

The peptides derived from the conserved epitopes were used as tagged peptides with either palmitate or Tat for better internalization in the cells. However, both of these molecules also interact with TLR [61,62] and therefore also act as an adjuvant in these studies. Previously, when scrambled peptides were delivered using the same strategy, animals receiving such mock vaccine became infected earlier than the control cats, suggesting palmitate or Tat addition enhances the infection (unpublished data). In order to prevent non-specific activation via TLR, future studies will use a minimally immunogenic or non-immunogenic viral vector with appropriately linked T-cell epitopes/genes to induce CD8^+^ T-cell immunity for vaccine prophylaxis with potential as a therapeutic vaccine. 

## 5. Conclusions

The systematic analysis for non-mutating epitopes, based on FIV/HIV lentiviral conservation for both anti-FIV/HIV activities and sequence similarity, most likely identified the highly conserved (mutation-resistant), protective FIV epitopes, as well as potential protective HIV epitopes. The robust FIV-specific T-cell immunogenicity and the significant MAP vaccine efficacy of the current MAP vaccine trial in cats support the assertion that anti-FIV/HIV T-cell epitope selection based on both polyfunctional T-cell and CTL activities successfully identified protective T-cell epitopes on FIV. In conclusion, this selection approach also identified potential protective HIV epitopes for an effective HIV vaccine while testing the epitope selection approach in the FIV/cat model for vaccine efficacy. 

## 6. Patents

United States Patent No. US 9,913,895 B2 and US 2018/0333481 AI (inventor JKY) has been published on March 13, 2018 and Nov. 22 2018, respectively from the work reported in this manuscript. 

## Figures and Tables

**Table 1:** Sequence conservation between the protective FIV peptides and their counterpart HIV-1 (HIV) peptides.

FIV or HIV Peptide ^ab^	FIV or Counterpart HIV Protein (FIV/HIV Subtype) ^cd^	Peptide AA No.^cde^	FIV or Counterpart HIV Peptide Sequence ^efg^	FIV and HIV % AA Similarity (% AA Identity) [F/H Gap] ^dfg^
Fp9-3	p24 (A,B,C,D)	15	FAPARMQCRAWYLEA	60% (13%) [H1] (44% (11%) [H1])
Hp10-3	p24 (B,A4,D,G,J,K)	15	NPPIPVGEIYKR-WII	62% (12%) [H1]
MAP4	p24 (A,B,C,D)	15	FAPARMQCRAWYLEA	60% (13%) [H1] (44% (11%) [H1])
Fp14-3	p24 (A,B,D)	14	AEVKLYLKQSLSIA	71% (29%) [0] (59% (24%) [0])
Fp14-4	p24 (A)	13	KLYLKQSLSIANA	77% (31%) [0] (67% (27%) [0])
Hp15-3/4	p24 (B,A6,D,K)	15	VKNWMTETLLVQNAN	80% (40%) [0]
MAP3	p24 (A,B,D)	16	AEVKLYLKQSLSIANA	75% (37%) [0] (65% (29%) [0])
FRT3-3	RT (B,D)	15	KKK-SGKWRMLIDFRV	75% (69%) [F1]
FRT3-4	RT (B,D)	13	WRMLIDFRVLNKL	77% (69%) [0] (71% (64%) [0])
HRT3-3	RT (B,A,C,D,F,G,H,J,K)	15	KKKDSTKWRKLVDFR	75% (69%) [F1]
HRT3-4	RT (B A,C,D,F,G,H,J,K)	14	KWRKLVDFRELNKR	79% (71%) [0]
MAP2	RT (B,D) ^h^	18	KKK-SGKWR-LIDFRVLNKL	75% (70%) [F2]
MAP2v	RT (B,D) ^h^	19	KKK-SGKWRLLIDFRVLNKL	75% (70%) [F1]
FRT7-1	RT (B,C,D)	15	GRRYVWCSLPQGWVL	60% (53%) [0]
FRT7-2	RT (B,C,D)	14	CSLPQGWVLSPLIY	64% (57%) [0]
HRT7-1	RT (B,A^i^,C,D,F,G,H,J)	15	GIRYQYNVLPQGWKG	60% (53%) [0]
HRT7-2	RT (B,A,C,D,F,G,H,J,K)	14	NVLPQGWKGSPAIF	64% (57%) [0]
MAP5	RT (B,C,D)	20	GRRYVWCSLPQGWVLSPLIY	65% (55%) [0]

^a^ FIV or HIV peptide start with (F) or (H); each MAP with FIV peptide(s); overlapping peptide Hp15-3/Hp15-4 (Hp15-3/4). ^b^ Peptide sequences used in the assays and the overlapping peptide used as the vaccine multiple antigenic peptide (MAP). ^c^ Original sequence from the first subtype listed; FIV subtype based on [31] and lists FIV subtypes with strains having identical sequence as the FIV peptides; HIV subtypes or sub-subtype (A4, A6) identical with at least 40% of subtype or sub-subtype sequences in the LANL QuickAlign database [32]. ^d^ Abbreviations: Reverse transcriptase (RT), amino acid (AA), number (No.), FIV gap number [F#], HIV gap number [H#]. ^e^ Peptide sequence and number without gap used for biological assays; those with gap (hyphen) used for sequence similarity/identity comparison. ^f^ Each peptide sequence compared to a section of the actual counterpart virus sequence. ^g^ FIV results with additional parenthesis (% similarity (% identity) [Gap]) are compared to the counterpart HIV peptide when the results differ between the HIV sequence and the closest counterpart HIV peptide or the overlapping HIV peptide. ^h^ Closest to subtypes B & D except for the deletion (-) or lysine (L) instead of methionine. ^i^ All sub-subtype A except for A3.

**Table 2:** Comparative immunogenicity analyses of FIV peptides to identify conserved protective T-cell epitopes.

FIV Peptide	% Responder Rate of Vaccinated Cats ^a^					Percent Responder Rate of HIV^+^ Human Subjects ^a^							In Silico HLA Analysis ^b^	
	Proliferation ^acd^		Cytokine ^ac^		FIV	Proliferation ^acd^		Perforin/GrzA/GrzB/CD107a ^cd^				Cytokine ^a^	NetMHC 4.0	NetMHC 2.3
	CD4 T	CD8 T	IFNγ	IL-2	E/S ^e^	CD4 T	CD8 T	CD4 CTL^a^	RF ^f^	CD8 CTL^a^	RF ^f^	IFNγ	HLA-ABC ^g^	DRB1
**Fp9-3**	66.7	**66.7**	66.7	0.0	S	27.8	**88.9**	**60.0**	**0.389 ^h^**	**70.0**	**0.444 ^h^**	9.1	A2, B8, B44 B27, C07, B83, C06	- ^i^
Fp14-3	33.3	**33.3**	33.3	33.3	-	0.0	**83.3**	70.0	**0.444 ^h^**	70.0	0.389 ^h^	66.7	A2, A24, A1, B44, C14	1201, 0405, 1302, 0404, 1602, 0401, 1001, 0101
**Fp14-4**	33.3	**33.3**	33.3	16.7	-	16.7	**100.0**	**100.0**	**0.444 ^h^**	**100.0**	**0.444 ^h^**	33.3	A2, A24, A1	0701, 1001, 1201, 0405, 1302, 0404, 0401, 1602, 0101
**FRT3-3**	42.9	**42.9**	14.3	14.3	E	0.0	**62.5**	66.7	**0.444**	83.3	0.278	100.0	B27, C15, A3, A1	1602, 1001, 0405, 0101, 1501
FRT3-4	42.9	**57.1**	28.6	42.9	S	0.0	37.5	66.7	**0.389**	66.7	**0.444**	10.0	B27, A1, B44, A3	0301, 1602, 0101, 0103, 0401, 0405, 1001,1501
**FRT7-1**	100.0	**100.0**	33.3	66.7	S ^j^	20.0	**20.0**	60.0	0.467	**100.0**	**0.667**	0.0	B27, A1, C07, A24, C06, C07, C08	0404
FRT7-2	66.7	**66.7**	33.3	0.0	S ^j^	0.0	0.0	**100.0**	**0.533**	**100.0**	**0.867**	0.0	A1, B27, A29, C15, A3, C08	1201
Average	55.1	57.1	34.7	24.8		6.4	56.4	74.8	0.444	84.3	0.505	31.3		
*p*-value ^k^		*0.8768*		*0.3898*			***0.0129***			*0.3060*	*0.4583*			

^a^ Percent responder rate (RR) is the number of responders to the peptide divided by the total number of responders multiplied by 100. ^b^ Human leukocyte antigen-A (HLA-A) and HLA-B in supertype, and HLA-C as C in 2-digit resolution nomenclature using the Net-MHC 4.0 algorithm for HLA-A, HLA-B, and HLA-C (HLA-ABC) [20]; 4-digit resolution nomenclature for HLA-DRB1 (DRB1) using the Net-MHCII 2.3 algorithm [22]; all in order from the strongest to the weakest binding potential. ^c^ CD4^+^ T-cell proliferation (CD4 T), CD8^+^ T-cell (CD8 T) proliferation, CD4^+^ cytotoxic T lymphocyte CTL (CD4 CTL), CD8^+^ CTL (CD8 CTL), granzyme A (GrzA), granzyme B (GrzB), CD107a in % fluorescence. ^d^ Bold CD8^+^ T-cell proliferation, CD4^+^ CTL, or CD8^+^ CTL with the two highest rates or response frequencies among the peptides from the same pool. ^e^ Peptide induced suppression (S), enhancement (E), or no effect (-) in the in vitro FIV infection assay. ^f^ Response frequency [RF] for CD4^+^ or CD8^+^ CTL shown as the total number of positive responses divided by the total number of possible responses. ^g^ Serotype for A29 (HLA-A2902). ^h^ Includes responder rate and response frequency for CD107a. ^i^ Strong binder with HLA-DRB3*0101 and weak binders with HLA-DRB1*1501, *0101, *0701, *1001, *1301, and *0301. ^j^ Suppression of in vitro FIV infection based on overlapping peptide FRT-7-1/FRT7-2 in MAP5. ^k^ Statistics between RRs of CD4^+^ and CD8^+^ T-cell proliferation, IL-2 and IFNγ production, CD4^+^ and CD8^+^ CTLs, and RFs of CD4^+^ and CD8^+^ CTLs. Significant in bold when *p* < 0.05.

**Table 3:** Summary of MAP immunization and challenge efficacy ^a^.

Group	Immunization ^ab^				No. of Protected/Total No. of Cats ^a^ [% Protection]						Preventable	*p-*value ^ad^
No. ^a^	Vaccine 1	Vaccine 2	Vaccine 3	Route	0wpc	3wpc	6wpc	9wpc	12wpc	16-24wpc	Fraction ^c^ (%)	1abc *vs.* 2a–d
**Individual Groups *versus* Combined Group 2a–d**												
1a	MAP2/3/4t	MAP2/2v/3/5	MAP2/2v/3/5	SC/ID	6/6	6/6	4/6	4/6	3/6	3/6	20%	0.6550
1b	MAP2v/3/4/5	MAP2v/3/4/5	MAP2v/3/4/5	SC	6/6	6/6	6/6	6/6	6/6	6/6	100%	0.0152
1c	MAP2v/3/4/5	MAP2v/3/4/5	MAP2v/3/4/5	ID	7/7	7/7	6/7	6/7	6/7	6/7	77.1%	0.0686
2a-d	Adj,PBS,-	Adj,PBS,-	Adj,PBS,-	SC/ID, SC, ID, -	16/16	15/16	8/16	6/16	6/16	6/16	-	-
2a	Adj	Adj	Adj	SC/ID	3/3	2/3	1/3	1/3	1/3	1/3	-	-
2b	PBS	PBS	PBS	SC/ID	5/5	5/5	4/5	3/5	3/5	3/5	-	-
2c	Adj	Adj	Adj	SC, ID	6/6	6/6	2/6	2/6	2/6	2/6	-	-
2d	-	-	-	-	2/2	2/2	1/2	0/2	0/2	0/2	-	-
**Combined Groups 1abc or 1bc *versus* Combined Group 2a–d**												
1abc	MAP2/3/4t MAP2v/3/4/5	MAP2/2v/3/5 MAP2v/3/4/5	MAP2/2v/3/5 MAP2v/3/4/5	SC/ID, SC, ID	19/19[100%]	19/19[100%]	16/19[84%]	16/19[84%]	15/19[79%]	15/19[79%]	66.3%	0.0180
1bc	MAP2v/3/4/5	MAP2v/3/4/5	MAP2v/3/4/5	SC, ID	13/13	13/13	12/13	12/13	12/13	12/13	87.7%	0.0057

^a^ Note: Detailed results are shown in Table S3. ^b^ Abbreviations: Number (No.); weeks post-challenge (wpc); bone marrow cells (BM); thymus cells (Th), mesenteric lymph node cells (mLN); vaccine with MAP2v, MAP3, MAP4, and MAP5 (MAP2v/3/4/5); vaccine with MAP2, MAP2v, MAP3, and MAP5 (MAP2/2v/3/5); vaccine with MAP2, MAP3, and MAP4t (MAP2/3/4t) where MAP4t with Tat instead of palmitic acid; adjuvant (Adj); subcutaneous (SC); intradermal (ID); concurrent SC and ID (SC/ID); not done or not applicable (-). ^c^ Adjuvant supplemented with feline IL-12 for ID and concurrent SC/ID vaccinations. ^d^ Percent preventable fraction equals the incidence of infected control group minus incidence of infected vaccine group divided by the incidence of infected control group and then multiplied by 100. ^e^ Combined control Group 2a-d compared to combined vaccine Group 1abc or 1bc with a significance at *p* < 0.05 in bold.

**Table 4:** Statistical comparison between unprotected/vaccinated cats and protected/vaccinated cats from Group 1bc or from all vaccine groups.

Unprotected *versus*	3-6w Post-2nd Vac ^a^		3w Post-3rd Vac ^a^		6wk Post-3rd Vac ^a^		3-6w Post-2nd Vac ^a^		3w Post-3rd Vac ^a^		6w Post-3rd Vac ^a^	
Protected (Table No.) ^b^	CD4 T ^cd^	CD8 T ^cd^	CD4 T ^cd^	CD8 T ^cd^	CD4 T ^cd^	CD8 T ^cd^	IL-2 ^cd^	IFNγ ^c^	IL-2 ^cd^	IFNγ ^cd^	IL-2 ^cd^	IFNγ ^cd^
**Percent responder rate ^e^**												
Group-1 unprotected (S5 *vs.* S6)	18.2	13.6	18.2	22.7	47.2 ^h^	41.1 ^h^	27.3	47.7	18.2	61.4	11.4	31.8
Group-1bc protected (S5 *vs.* S6)	**40.6**	**27.3**	**65.7**	**55.9**	**67.1 ^h^**	**59.4 ^h^**	**41.3**	38.5	**24.5**	27.3	**21.7**	26.6
*p-value*^g^ (S5 *vs.* S6)	***0.0078***	*0.0648*	***0.00003***	***0.0018***	*0.2308*	*0.1353*	*0.2821*	*0.4747*	*0.4599*	***0.0173***	*0.2589*	*0.6396*
Group-1 unprotected (5)	18.2	15.9	18.2	22.8	48.0 ^i^	40.2 ^i^	27.3	47.8	18.2	61.3	11.4	31.8
Group-1 protected (5)	**39.2**	**30.1**	**59.1**	**55.1**	**67.7 ^i^**	**56.6 ^i^**	**38.6**	38.1	**22.7**	25.6	**18.2**	24.4
*p-value*^g^ (5)	*0.0732*	***0.0335***	***0.0038***	***0.0004***	***0.0125***	*0.3079*	*0.1374*	*0.2754*	*0.6435*	***0.0041***	*0.4511*	*0.6441*
**Average magnitude of the positive responses ^f^**												
Group-1 unprotected (S5 *vs.* S6)	2.8	7.6	2.6	14.8	3.2 ^h^	5.6 ^h^	70.6	181.4	53.6	228.4	34.6	127.9
Group-1bc protected (S5 *vs.* S6)	5.8	7.5	**10.6**	9.0	**10.8 ^h^**	**7.6 ^h^**	**125**	142.3	**153.6**	**240.3**	**175**	**172.3**
*p-value*^g^ (S5 *vs.* S6)	*0.1014*	*0.9992*	***0.0083***	*0.5740*	***0.0471***	*0.5698*	*0.0781*	*0.3902*	***0.0266***	*0.8993*	***0.0072***	*0.4951*
Group-1 unprotected (5)	3.4	22.5	2.8	27.8	5.3 ^i^	6.7 ^i^	158.3	254.5	68.6	262.2	54.0	117.4
Group-1 protected (5)	**4.5**	14.2	**9.3**	8.2	**9.8** **^i^**	**8.1 ^i^**	137.7	159.7	**112.1**	156.2	**129.6**	**145.5**
*p-value*^g^ (5)	*0.6626*	*0.4710*	***0.0022***	*0.3311*	*0.0529*	*0.5551*	*0.5263*	*0.2959*	*0.3436*	*0.2230*	*0.1257*	*0.6801*

^a^ At 3, 6, or 3–6 weeks (w) post-2nd or -3rd vaccination (Vac). ^b^ Data derived from the designated Table(s). ^c^ CD4^+^ T-cell (CD4 T) or CD8^+^ T-cell (CD8 T) proliferation; IL-2 and IFNγ production. ^d^ Bold average value from the protected group when larger than the one from the unprotected group. ^e^ Percent responder rate: The number of responders to the peptide divided by the total number of responders multiplied by 100. ^f^ Magnitude of CD4^+^ and CD8^+^ T-cell proliferation in average % CFSE^low^; magnitude of IFNγ and IL-2 production in average SFU/10^6^ PBMC. ^g^ Statistics between the unprotected group and all protected cats or the protected cats in Group 1bc at each time-point with a significance at *p*<0.05 in bold. ^h^ Missing MAP4 and Fp9-3 from Group 1a, therefore the average values excluded from all results for MAP4 and Fp9-3 for both unprotected and protected cats. ^i^ Missing MAP4 and Fp9-3 from Group 1a, but included the MAP4 and Fp9-3 results from Group 1bc.

**Table 5:** Responder rate and average magnitude of the immune responses of individual vaccinated cats at six weeks post-third vaccination.

	Protected/Vaccinated Cats (P)																Unprotected Cats (U)				
Immune	Vaccine Group 1a ^a^			Vaccine Group 1b ^a^							Vaccine Group 1c (G1c) ^a^						Vaccine Group 1a ^a^			G1c^ab^	U *vs.* P ^b^
Anal. ^b^	BFA	DU4	DX5	HOC	RL2	RN4	RO1	RO7	RP4	RQ1	HOB	RL4	RN3	RO2	RP3	RQ5	DU5	DX1	OLL	RL1	*p*-value ^c^
**Percent responder rate^d^**																					
**CD8 T**	44.4 ^f^	33.3 ^f^	55.6 ^f^	72.7	81.8	63.6	63.6	100.0	100.0	63.6	54.5	0.0	18.2	36.4	45.5	72.7	11.1 ^f^	55.6 ^f^	66.7 ^f^	27.3	*0.3079*
**CD4 T**	77.8 ^f^	55.6 ^f^	77.8 ^f^	63.6	36.4	72.7	81.8	90.9	81.8	90.9	54.5	54.5	63.6	45.5	81.8	54.5	55.6 ^f^	55.6 ^f^	44.4 ^f^	36.4	***0.0125***
**Average**	61.1	44.5	66.7	68.2	59.1	68.2	72.7	95.5	90.9	77.3	54.5	27.3	40.9	41.0	63.7	63.6	33.4	55.6	55.6	31.9	
**IL-2**	9.1	0.0	0.0	0.0	9.1	54.5	18.2	9.1	18.2	54.5	0.0	0.0	36.4	0.0	36.4	45.5	0.0	0.0	18.2	27.3	*0.4511*
**IFNγ**	36.4	0.0	9.1	0.0	0.0	36.4	36.4	9.1	0.0	9.1	63.6	0.0	54.5	45.5	36.4	54.5	0.0	18.2	54.5	54.5	*0.6441*
**Average**	22.8	0.0	4.6	0.0	4.6	45.5	27.3	9.1	9.1	31.8	31.8	0.0	45.5	22.8	36.4	50.0	0.0	9.1	36.4	40.9	
**Average magnitude of positive responses^e^**																					
**CD8 T**	21.2	6.7	6.2	6.9	3.1	9.5	2.6	9.6	9.3	6.6	6.2	0.0	11.3	3.7	10.4	16.2	2.0	9.2	9.5	6.2	*0.5551*
**CD4 T**	3.9	1.6	2.3	14.4	7.9	11.7	10.9	10.3	10.9	20.1	7.0	4.1	11.6	4.8	15.6	20.1	9.2	3.3	2.7	5.9	*0.0529*
**Average**	12.5	4.1	4.2	10.7	5.5	10.6	6.7	10.0	10.1	13.3	6.6	2.1	11.4	4.3	13.0	18.1	5.6	6.2	6.1	6.0	
**IL-2**	51	0	0	0	372	205	373	105	238	214	0	0	144	0	255	119	0	0	105	111	*0.1257*
**IFNγ**	78	0	56	0	0	96	133	116	0	546	382	0	237	179	362	144	0	63	192	215	*0.6801*
**Average**	33	0	28	0	186	150	253	111	119	380	191	0	190	90	309	132	0	31	148	163	

^a^ Individual cat designated with identification code distributed into protected/vaccinated and unprotected/vaccinated groups. ^b^ Abbreviations: Analyses (Anal.), CD4^+^ T-cell proliferation (CD4 T), CD8^+^ T-cell proliferation (CD8 T), Vaccine Group 1c (G1c), unprotected (U), protected (P). ^c^ Statistics between all unprotected cats and all protected cats for each immune analysis, with a significance at *p*<0.05 in bold. ^d^ Percent responder rate: The number of responders to the peptide divided by the total number of responders multiplied by 100. ^e^ Magnitude of CD4^+^ and CD8^+^ T-cell proliferation in average % CFSE^low^; magnitude of IFNγ and IL-2 production in average SFU/10^6^ PBMC. ^f^ Analysis performed without results of MAP4 and Fp9-3 at six weeks post-third vaccination.

## Supplementary Materials

The following are available online at http://www.mdpi.com/1999-4915/11/2/136/s1: Figure S1. FIV MAP/peptide-specific T-cell proliferation and cytokine production of the PBMC from vaccinated cats at 3-6 weeks post-second vaccination, Figure S2. FIV MAP/peptide-specific T-cell proliferation and cytokine production of the PBMC from vaccinated cats at six weeks post-third vaccination, Figure S3. qRT-PCR analysis for the mRNA levels to the cytokines and the CTL-associated cytolysin/cytotoxins in the PBMC from vaccinated cats at six weeks post-third vaccination, Table S1. Selection of FIV p24 and RT peptide pool based on the number of responders, Table S2. The number of responders to individual peptides from the FIV peptide pools with the most feline or human CTL or CD8^+^ T-cell responders, Table S3. Challenge efficacy determined by virus load, proviral PCR, and FIV antibody detection, Table S4. Immunogenicity of HIV peptides counterpart to protective FIV peptides and FRT11 peptides, Table S5. Responder rate and average magnitude of the MAP/peptide responses by the unprotected cats from all vaccine groups, Table S6. Responder rate and average magnitude of the MAP/peptide responses by the protected cats from vaccine Group 1bc, Table S7. Comparing the responder rate between the selection study and MAP vaccine trial and between the post-second and post-third vaccinations, Table S8. Primers and probes for qRT-PCR.
