# Supplementary material for: Immunogenicity and Efficacy of a Novel Multi-Antigenic Peptide Vaccine Based on Cross-Reactivity between Feline and Human Immunodeficiency Viruses
Source: Viruses. 2019 Feb 3;11(2):136. doi: 10.3390/v11020136 (PMC6409633; doi:10.3390/v11020136)
Supplement: Supplementary file 1 [file viruses-11-00136-s001.pdf]

Supplemental Figure Legends and Figures

Figure S1. FIV MAP/peptide-specific T-cell proliferation and cytokine production of the PBMC from vaccinated cats at 3-6 weeks post-2nd vaccination. The results from $CD4^+$ (A) and $CD8^+$ (B) T-cell proliferation and the IL-2 (C) and IFNγ (D) production of the PBMC from the MAP-vaccinated cats are shown in response to the in vitro stimulation with MAP or FIV peptide. The vaccinated cats in Group 1a received concurrent subcutaneous plus intradermal (SC/ID) vaccinations with triple-MAP vaccine (grey bars) and evaluated for immune responses at 3 weeks post-2nd vaccination. Those in Groups 1b and 1c received either subcutaneous (SC) (dark blue bars) or intradermal (ID) (blue bars) vaccinations with quadruplicate-MAP vaccine, respectively and evaluated for immune responses at 6 weeks post-2nd vaccination. The identification codes of each vaccinated cat are shown in the top right inset of panel A. Each bar represents the average response from a single vaccinated cat after subtraction of the average value of the results from eight control cats, with the exception of the mitogen control. Only bars at ≥0.5% $CFSE^{low}$ threshold for $CD4^+$ and $CD8^+$ T-cell proliferation or at ≥50 SFU/10^6 PBMC threshold for IL-2 and IFNγ production are shown.

Figure S2. FIV MAP/peptide-specific T-cell proliferation and cytokine production of the PBMC from vaccinated cats at 6 weeks post-3rd vaccination. The results from $CD4^+$ (A) and $CD8^+$ (B) T-cell proliferation and the IL-2 (C) and IFNγ (D) production of the PBMC from the MAP-vaccinated cats are shown in response to the in vitro stimulation with MAP or FIV peptide. The vaccinated cats in Group 1a received concurrent subcutaneous plus intradermal (SC/ID) vaccinations with triple-MAP vaccine (grey bars), while those in Groups 1b and 1c received either subcutaneous (SC) (dark blue bars) or intradermal (ID) (blue bars) vaccinations with quadruplicate-MAP vaccine, respectively. The identification codes of each vaccinated cat are shown in the top right inset of panel A. Vaccinated cats in Group 1a were not tested (NT) for $CD4^+$ and $CD8^+$ T-cell proliferation to MAP4 and Fp9-3 as shown with (NT) in panels A and B. Each bar represents the average response from a single vaccinated cat after subtraction of the average value of the results from eight control cats, with the exception of the mitogen control. Only bars at ≥0.5% $CFSE^{low}$ threshold for $CD4^+$ and $CD8^+$ T-cell proliferation or at ≥50 SFU/10^6 PBMC threshold for IL-2 and IFNγ production are shown.

Figure S3. qRT-PCR analysis for the mRNA levels to the cytokines and the CTL-associated cytolysin/cytotoxins in the PBMC from vaccinated cats at 6 weeks post-3rd vaccination. The PBMC from four vaccinated cats each in Groups 1b (white bar) and 1c (black bar) and from four adjuvant control cats in Group 2c were stimulated for 14 h with either MAP, Fp9-3 peptide, or FIV peptide mixture. The FIV peptide mixtures consisted of FRT3-3/4 (FRT3-3, FRT3-4, and overlapping FRT3-3/FRT3-4 peptides), Fp14-3/4 (Fp14-3, Fp14-4, and overlapping Fp14-3/Fp14-4 peptides), and FRT7-1/2 (FRT7-1, FRT7-2, overlapping FRT7-1/FRT7-2 peptides). The mRNA levels of cytokines IL-2 and IFNγ, cytolysin perforin, cytotoxins GrzA and GrzB, and the surface cell marker CD107a molecule are shown as the relative expression of the average of four cats each from vaccine Groups 1b and 1c relative to the adjuvant control group. The results from the two vaccine groups are normalized with internal control GAPDH, and then represented as fold change over their media controls and the adjuvant control group. Those bars with more than two-fold difference (above dotted line) are considered significant. Significant difference (p<0.05) between the two vaccine Groups 1b and 1c are shown with (*) above the Group 1c bar. Note that those bars without standard deviation bar are the results from one cat such as the GrzB results shown with responses from one cat each in both groups with exception of the response to FRT3-3/4 by Group 1c.

Fig S1

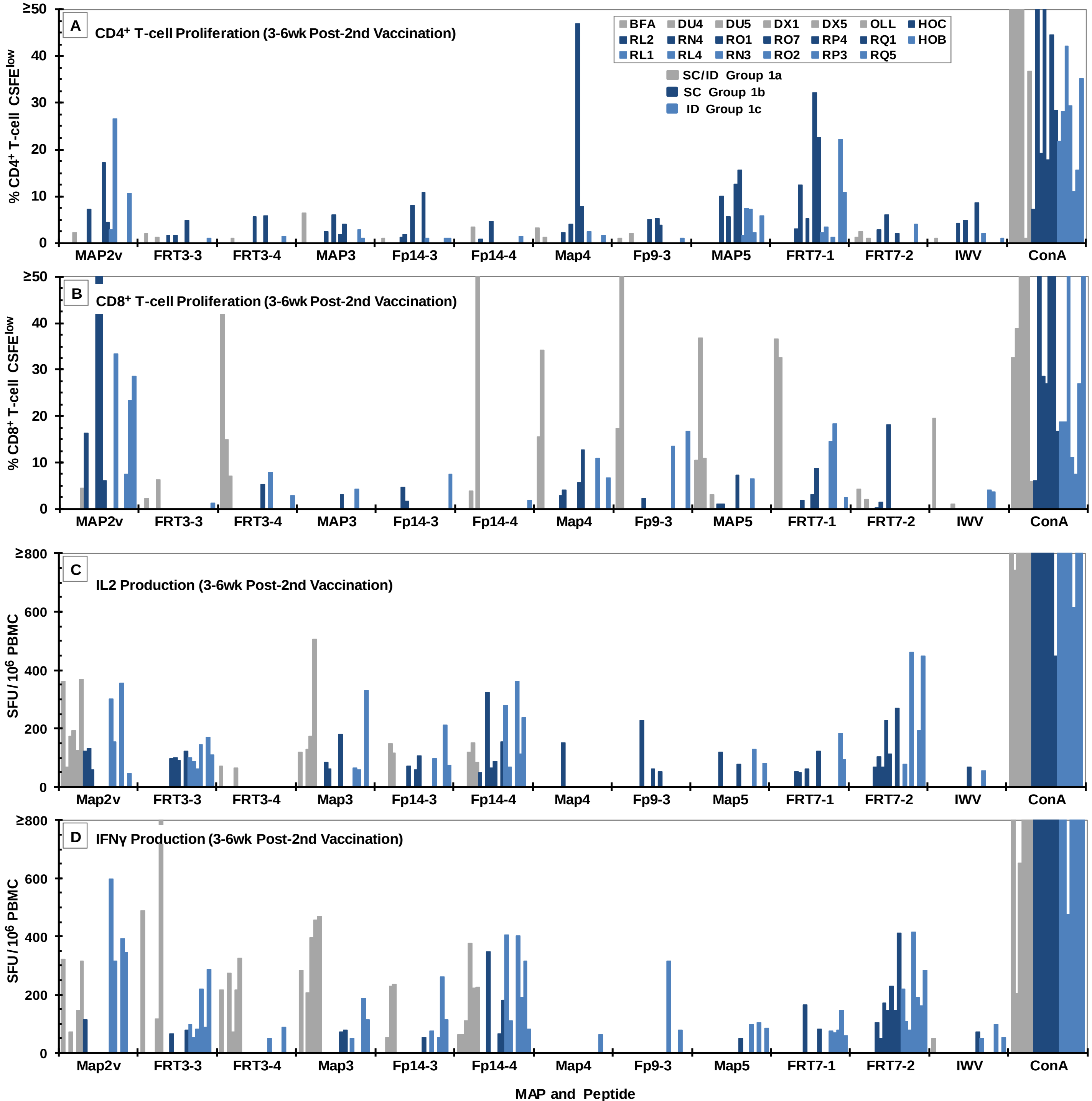
A
CD4+ T-cell Proliferation (3-6wk Post-2nd Vaccination)
% CD4+ T-cell CSFE low
≥50
40
30
20
10
0
BFA
DU4
DU5
DX1
DX5
OLL
HOC
RL2
RN4
RO1
RO7
RP4
RQ1
HOB
RL1
RL4
RN3
RO2
RP3
RQ5
SC/ID Group 1a
SC Group 1b
ID Group 1c
MAP2v
FRT3-3
FRT3-4
MAP3
Fp14-3
Fp14-4
Map4
Fp9-3
MAP5
FRT7-1
FRT7-2
IWV
ConA
B
CD8+ T-cell Proliferation (3-6wk Post-2nd Vaccination)
% CD8+ T-cell CSFE low
C
IL2 Production (3-6wk Post-2nd Vaccination)
SFU / 10^6 PBMC
≥800
600
400
200
0
Map2v
Map3
Map5
D
IFNγ Production (3-6wk Post-2nd Vaccination)
MAP and Peptide

Fig S2

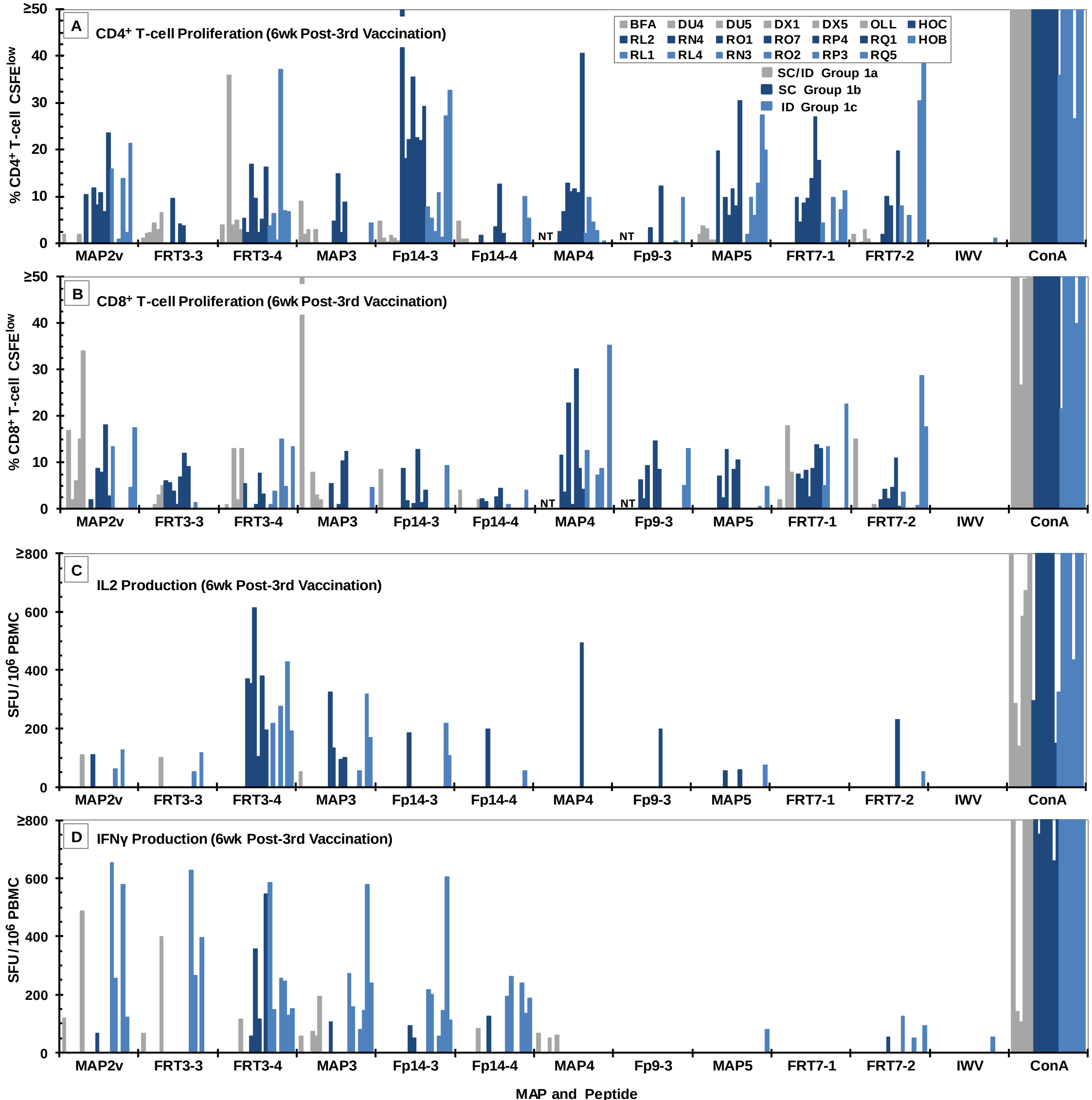
A
CD4+ T-cell Proliferation (6wk Post-3rd Vaccination)
% CD4+ T-cell CSFElow
B
CD8+ T-cell Proliferation (6wk Post-3rd Vaccination)
% CD8+ T-cell CSFElow
C
IL2 Production (6wk Post-3rd Vaccination)
D
IFNγ Production (6wk Post-3rd Vaccination)
SFU / 10^6 PBMC
BFA DU4 DU5 DX1 DX5 OLL HOC
RL2 RN4 RO1 RO7 RP4 RQ1 HOB
RL1 RL4 RN3 RO2 RP3 RQ5
SC/ID Group 1a
SC Group 1b
ID Group 1c
≥50
≥800
NT
MAP2v
FRT3-3
FRT3-4
MAP3
Fp14-3
Fp14-4
MAP4
Fp9-3
MAP5
FRT7-1
FRT7-2
IWV
ConA
MAP and Peptide

Fig S3

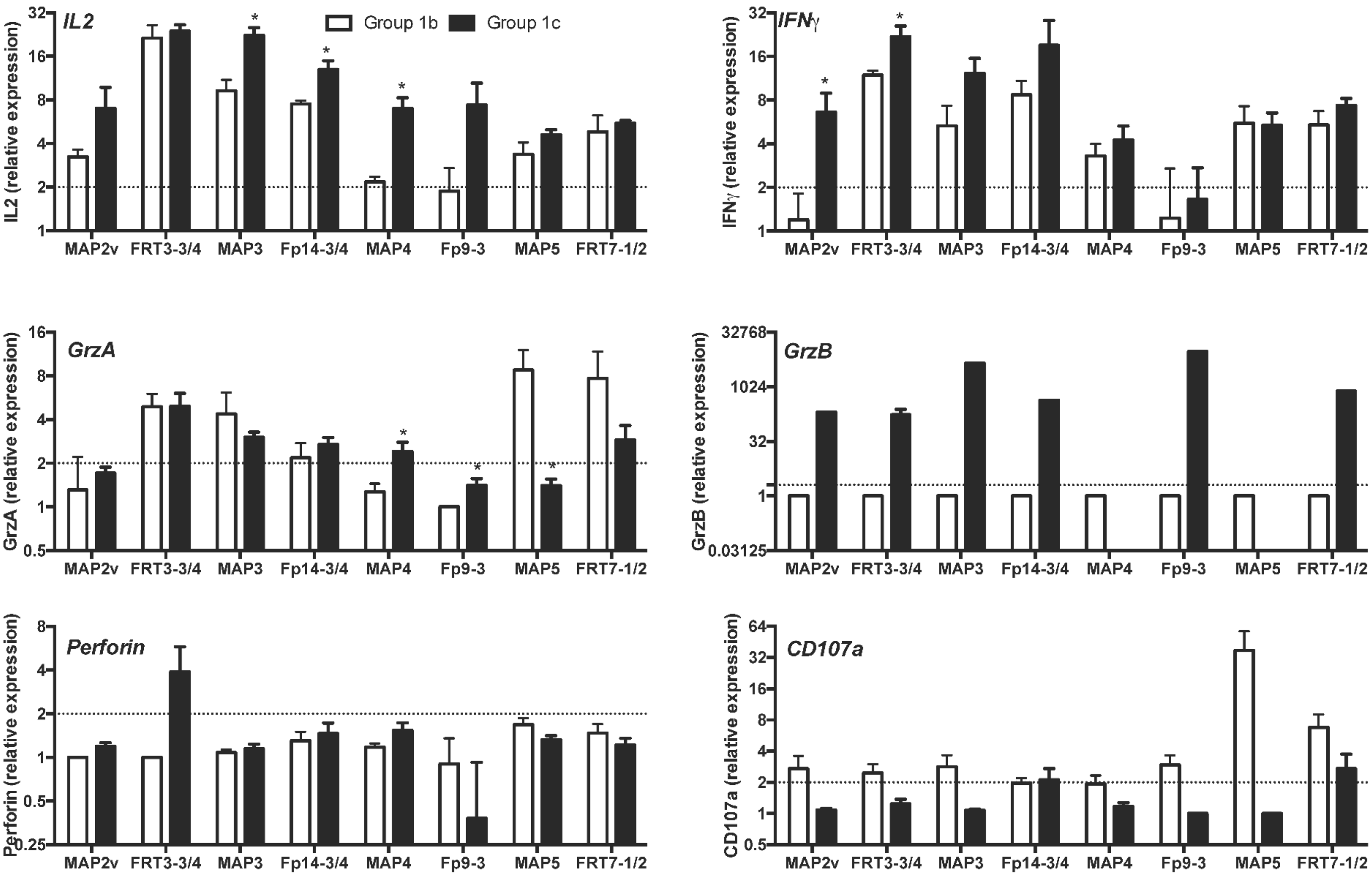

IL2
Group 1b
Group 1c
IL2 (relative expression)
IFNγ
IFNγ (relative expression)
GrzA
GrzA (relative expression)
GrzB
GrzB (relative expression)
Perforin
Perforin (relative expression)
CD107a
CD107a (relative expression)
MAP2v
FRT3-3/4
MAP3
Fp14-3/4
MAP4
Fp9-3
MAP5
FRT7-1/2

Supplemental Tables S1-S8

Table S1. Selection of FIV p24 and RT peptide pool based on the number of responders

Table S2. Number of responders to individual peptides from the FIV peptide pools with the most feline or human CTL or $CD8^+$ T-cell responders

Table S3. Challenge efficacy determined by virus load, proviral PCR, and FIV antibody detection

Table S4. Immunogenicity of HIV peptides counterpart to protective FIV peptides and FRT11 peptides

Table S5. Responder rate and average magnitude of the MAP/peptide responses by the unprotected cats from all vaccine groups

Table S6. Responder rate and average magnitude of the MAP/peptide responses by the protected cats from vaccine Group 1bc

Table S7. Comparing the responder rates between the selection study and MAP vaccine trial and between the post-2nd and post-3rd vaccinations

Table S8. Primers and probes for qRT-PCR

Table S1. Selection of FIV p24 and RT peptide pool based on the number of responders

FIV Peptide Pool [b]	Responder Rate of FIV-vaccinated Cats (%) [a]				Responder Rate of HIV$^+$ Human Subjects (%) [a]				
	Proliferation		Cytokine Production		Proliferation		Perforin/GrzA/GrzB		Cytokine
	CD4 T	CD8 T	IFNγ	IL-2	CD4 T	CD8 T	CD4 CTL	CD8 CTL	IFNγ
**Fp3** [c]	9/18 (50)	9/18 (50)	7/12 (58)	0/6 (0)	0/27 (0)	0/27 (0)	n	n	**2/21 (10)**
**Fp4** [c]	11/18 (61)	8/18 (44)	1/12 (8)	2/6 (33)	0/27 (0)	0/27 (0)	n	n	**2/21 (10)**
Fp5	9/18 (50)	7/18 (39)	4/12 (33)	2/6 (33)	1/27 (4)	0/27 (0)	n	n	1/21 (5)
**Fp9** [c]	6/18 (33)	**9/18 (50)**	2/12 (17)	0/6 (0)	1/27 (4)	**7/27 (26)**	**8/8 (100)**	**7/8 (87)**	0/21 (0)
**Fp10** [c]	7/18 (39)	3/18 (17)	5/12 (42)	6/6 (100)	1/27 (4)	0/27 (0)	2/5 (40)	2/5 (40)	**2/21 (10)**
**Fp14** [c]	13/18 (72)	**10/18 (55)**	4/12 (33)	5/6 (83)	2/27 (7)	**4/27 (15)**	**5/5 (100)**	**4/5 (80)**	**7/21 (33)**
Fp16	8/18 (44)	9/18 (50)	0/12 (0)	4/6 (67)	0/27 (0)	0/27 (0)	n	n	1/21 (5)
**FRT3** [c]	2/11 (18)	4/11 (36)	3/12 (25)	3/6 (50)	3/21 (14)	**12/26 (46)**	**5/11 (45)**	**7/11 (64)**	**18/32 (56)**
FRT4	8/11 (73)	4/11 (36)	5/12 (42)	6/6 (100)	1/21 (5)	0/26 (0)	n	n	4/32 (12)
FRT6	4/11 (36)	8/11 (73)	0/12 (0)	0/6 (0)	1/21 (5)	6/26 (23)	2/6 (33)	3/6 (50)	6/32 (19)
**FRT7** [c]	6/11 (55)	**7/11 (64)**	4/12 (33)	5/6 (83)	1/21 (5)	**7/26 (27)**	**2/5 (40)**	**3/5 (60)**	5/32 (16)
FRT14	5/11 (45)	5/11 (45)	5/12 (42)	6/6 (100)	2/21(10)	5/26 (19)	n	n	5/32 (16)
FRT15	3/11 (27)	4/11 (36)	3/12 (25)	1/6 (100)	5/21 (24)	6/26 (23)	n	n	3/32 (9)
FRT16	6/11 (55)	5/11 (45)	6/12 (50)	6/6 (100)	2/21 (10)	2/26 (8)	n	n	5/32 (16)
FRT20	6/11 (55)	5/11 (45)	5/12 (42)	6/6 (100)	2/21 (10)	3/26 (12)	n	n	5/32 (16)
FRT21	5/11 (45)	5/11 (45)	3/12 (25)	5/6 (83)	3/21 (14)	6/26 (23)	n	n	1/32 (3)

[a] Abbreviations: CD4$^+$ T cell (CD4 T), CD8$^+$ T cell (CD8 T), CD4$^+$ CTL (CD4 CTL), CD8$^+$ CTL (CD8 CTL), granzyme A (GrzA), granzyme B (GrzB), not done (n).

[b] FIV peptide pools for p24 (Fp#) and RT (FRT#) selected from previous studies using HIV$^+$ human subjects [17,18].

[c] Bolded peptide pools with high functional values (bold) to be tested in Table S2 for individual peptides with high functions and HIV reactivity.

Table S2. Number of responders to individual peptides from the FIV peptide pools with the most feline or human CTL or $CD8^+$ T-cell responders

**FIV Pool or Peptide** [c]	**Responder Rate of FIV-vaccinated Cats (%)** [a]				**Responder Rate of HIV^+ Human Subjects (%) [Response Frequency]** [ab]				
	**Proliferation**		**Cytokine Production**		**Proliferation**		**Perforin/GrzA/GrzB/*CD107a**		**Cytokine**
	**CD4 T**	**CD8 T** [d]	**IFNγ**	**IL-2**	**CD4 T**	**CD8 T** [d]	**CD4 CTL** [d]	**CD8 CTL** [d]	**IFNγ** [d]
Fp3 Pool	9/18 (50)	9/18 (50)	7/12 (58)	0/6 (0)	0/27 (0)	0/27 (0)	n	n	2/21 (10)
Fp3-1	1/6 (17)	1/6 (17)	1/6 (17)	1/6 (17)	n	n	n	n	n
Fp3-2	3/6 (50)	2/6 (33)	0/6 (0)	1/6 (17)	n	n	n	n	n
Fp3-3	2/6 (33)	2/6 (33)	0/6 (0)	0/6 (0)	n	n	n	n	n
Fp3-4	0/6 (0)	**3/6 (50)**	1/6 (17)	2/6 (33)	n	n	n	n	n
Fp4 Pool	11/18 (61)	8/18 (44)	1/12 (8)	2/6 (33)	0/27 (0)	0/27 (0)	n	n	2/21 (10)
Fp4-1	2/6 (33)	**2/6 (33)**	0/6 (0)	2/6 (33)	n	n	n	n	n
Fp4-2	0/6 (0)	1/6 (17)	2/6 (33)	3/6 (50)	n	n	n	n	n
Fp4-3	0/6 (0)	1/6 (17)	3/6 (50)	1/6 (17)	n	n	n	n	n
Fp9 Pool	6/18 (33)	9/18 (50)	2/12 (17)	0/6 (0)	3/33 (9)	9/33 (27)	8/8 (100) [0.542]	7/8 (87) [0.542]	0/21 (0)
Fp9-1	n	n	n	n	2/13 (15)	5/13 (38)	2/5 (40) [0.278]	**4/5 (80)** [0.389]	2/4 (50)
Fp9-2	0/1 (0)	0/1 (0)	0/1 (0)	1/1 (100)	2/13 (15)	6/13 (46)	**3/5 (60)** [0.333]	3/5 (60) [0.333]	3/4 (75)
**Fp9-3** [e]	2/3 (67)	**2/3 (67)***	2/3 (67)	0/3 (0)	5/18 (28)	**16/18 (89)***	**6/10 (60) [0.389]***	7/10 (70) **[0.444]***	1/11 (9)
Fp10 Pool	7/18 (39)	3/18 (17)	5/12 (42)	6/6 (100)	1/27 (4)	0/27 (0)	2/5 (40) [0.333]	2/5 (40) [0.267]	2/21 (10)
Fp10-1	0/6 (0)	1/6 (17)	3/6 (50)	1/6 (17)	5/13 (38)	3/13 (23)	2/5 (40) [0.333]	**3/5 (60) [0.400]**	0/5 (0)
Fp10-2	3/9 (33)	**5/9 (56)**	3/9 (33)	3/9 (33)	4/13 (31)	**5/13 (38)**	2/5 (40) [0.267]	2/5 (40) [0.200]	0/5 (0)
Fp10-3	3/9 (33)	**6/9 (67)**	2/9 (22)	2/9 (22)	1/13 (8)	3/13 (23)	**4/5 (80) [0.400]**	**3/5 (60)** [0.267]	1/5 (20)
Fp14 Pool	13/18 (72)	10/18 (55)	4/12 (33)	5/6 (83)	2/27 (7)	4/27 (15)	5/5 (100) [0.833]	4/5 (80) [0.667]	**7/21 (33)**
Fp14-1	2/6 (33)	1/6 (17)	3/6 (50)	4/6 (67)	2/10 (20)	4/10 (40)	4/5 (80) [0.389]	4/5 (80) **[0.444]**	3/9 (33)
Fp14-2	2/6 (33)	2/6 (12)	2/6 (33)	3/6 (50)	0/6 (0)	3/6 (50)	3/5 (60) [0.333]	4/5 (80) **[0.444]**	1/9 (11)
**Fp14-3** [e]	2/6 (33)	**2/6 (33)***	2/6 (33)	2/6 (33)	0/6 (0)	**5/6 (83)***	7/10 (70) **[0.444]***	7/10 (70) [0.389]	**6/9 (67)***
**Fp14-4** [e]	2/6 (33)	**2/6 (33)***	2/6 (33)	1/6 (17)	1/6 (17)	**6/6 (100)***	**10/10 (100)[0.444]***	**10/10 (100)[0.444]***	3/9 (33)
FRT3 Pool	2/11 (18)	4/11 (36)	3/12 (25)	3/6 (50)	3/21 (14)	12/26 (46)	5/11 (45) [0.455]	7/11 (64) [0.636]	**18/32 (56)**
FRT3-1	0/6 (0)	1/6 (17)	1/6 (17)	3/6 (50)	1/4 (25)	2/8 (25)	**5/6 (83)** [0.333]	**6/6 (100)[0.444]**	1/10 (10)
FRT3-2	2/7 (29)	2/7 (29)	1/7 (14)	1/7 (14)	0/4 (0)	3/8 (37)	4/6 (67) [0.278]	5/6 (83) [0.389]	0/10 (0)
**FRT3-3** [e]	3/7 (43)	**3/7 (43)***	1/7 (14)	1/7 (14)	0/4 (0)	**5/8 (62)***	4/6 (67) **[0.444]***	5/6 (83) [0.278]	**10/10 (100)***
**FRT3-4** [e]	3/7 (43)	**4/7 (57)***	2/7 (29)	3/7 (43)	0/4 (0)	3/8 (37)	4/6 (67) [0.389]	4/6 (67) **[0.444]***	1/10 (10)
FRT3-5	0/7 (0)	2/7 (29)	1/7 (14)	2/7 (29)	0/4 (0)	3/8 (37)	3/6 (50) [0.222]	4/6 (67) [0.389]	0/10 (0)
FRT7 Pool	6/11 (55)	7/11 (64)	4/12 (33)	5/6 (83)	1/21 (5)	7/26 (27)	2/5 (40) [0.400]	3/5 (60) [0.333]	5/32 (16)
**FRT7-1** [e]	3/3 (100)	**3/3 (100)***	1/3 (33)	2/3 (67)	1/5 (20)	**1/5 (20)**	3/5 (60) [0.467]	**5/5 (100)[0.667]***	0/5 (0)
**FRT7-2** [e]	2/3 (67)	**2/3 (67)***	1/3 (33)	0/3 (0)	0/5 (0)	0/5 (0)	**5/5 (100) [0.533]***	**5/5 (100)[0.867]***	0/5 (0)
FRT7-3	n	n	n	n	0/5 (0)	0/5 (0)	3/5 (60) [0.400]	2/5 (40) [0.267]	1/5 (20)
FRT7-4	n	n	n	n	0/5 (0)	0/5 (0)	**5/5 (100)** [0.400]	3/5 (60) [0.533]	0/5 (0)

[a] Abbreviations: $CD4^+$ (CD4 T) and $CD8^+$ T cell (CD8 T), $CD4^+$ CTL (CD4 CTL), $CD8^+$ CTL (CD8 CTL), granzyme A (GrzA), granzyme B (GrzB), not done (n), tested also for CD107a (*).

[b] Individual peptide results are generally higher due to the higher numbers of HIV^+ subjects with peptide-pool positive responders.

[c] FIV peptide pools for p24 (Fp#) and RT (FRT#) with individual peptides as pool number-overlapping peptide number (Fp#-# or FRT#-#).

[d] Bold values represent the highest one or two results among the individual peptide pool.

[e] Bold peptides selected for MAP formulation and tested as MAP vaccine in cats based on their high functional values in bold with (*).

Table S3. Challenge efficacy determined by virus load, proviral PCR, and FIV antibody detection

Sub-group - Cat Code[c]	Immunization: Vaccine[adef]	Immunization: Route[af]	Proviral PCR / Virus Load / FIV antibodies[ab]: 0 wpc	3 wpc	6 wpc	9 wpc	12 wpc	16 wpc	24 wpc	Tissue Load[ab]: BM	Th	mLN	Sub-group Rate[c]	Groups 1 & 2 Protect. Rate[ac]
1a- BFA	MAP2/±2v/3/±4t/±5	SC/ID	-/-/-	-/-/-	-/-/-	-/-/-	-/-/-	-/-/-	-/-/-	0	0	0	3/6	15/19
1a-DU4	MAP2/±2v/3/±4t/±5	SC/ID	-/-/-	-/-/-	-/-/-	-/-/-	-/-/-	-/-/-	-/-/-	n	n	n		
1a-DU5	MAP2/±2v/3/±4t/±5	SC/ID	-/-/-	-/-/-	+/2.5/-	+/3.0/+	+/3.0/+	+/6.0/+	+/5.0/+	6.0	6.0	2.5		
1a-DX1	MAP2/±2v/3/±4t/±5	SC/ID	-/-/-	-/-/-	+/2.0/-	+/3.0/+	+/3.0/+	+/4.0/+	+/3.5/+	5.0	5.5	3.5		
1a-DX5	MAP2/±2v/3/±4t/±5	SC/ID	-/-/-	-/-/-	-/-/-	-/-/-	-/-/-	-/-/-	-/-/-	n	n	n		
1a-OLL	MAP2/±2v/3/±4t/±5	SC/ID	-/-/-	-/-/-	-/-/-	+/-/-	+/-/+	+/3.0/+	+/1.5/+	3.5	2.0	0.5		
1b-HOC	MAP2v/3/4/5	SC	-/-/-	-/-/-	-/-/-	-/-/-	-/-/-	-/-/-	-/-/-	n	n	n	6/6	
1b-RL2	MAP2v/3/4/5	SC	-/-/-	-/-/-	-/-/-	-/-/-	-/-/-	-/-/-	-/-/-	n	n	n		
1b-RN4	MAP2v/3/4/5	SC	-/-/-	-/-/-	-/-/-	-/-/-	-/-/-	-/-/-	-/-/-	n	n	n		
1b-RO7	MAP2v/3/4/5	SC	-/-/-	-/-/-	-/-/-	-/-/-	-/-/-	-/-/-	-/-/-	n	n	n		
1b-RP4	MAP2v/3/4/5	SC	-/-/-	-/-/-	-/-/-	-/-/-	-/-/-	-/-/-	-/-/-	n	n	n		
1b-RQ1	MAP2v/3/4/5	SC	-/-/-	-/-/-	-/-/-	-/-/-	-/-/-	-/-/-	-/-/-	n	n	n		
1c-HOB	MAP2v/3/4/5	ID	-/-/-	-/-/-	-/-/-	-/-/-	-/-/-	-/-/-	-/-/-	0	0	0	6/7	
1c-RL4	MAP2v/3/4/5	ID	-/-/-	-/-/-	-/-/-	-/-/-	-/-/-	-/-/-	-/-/-	n	n	n		
1c-RN3	MAP2v/3/4/5	ID	-/-/-	-/-/-	-/-/-	-/-/-	-/-/-	-/-/-	-/-/-	n	n	n		
1c-RO2	MAP2v/3/4/5	ID	-/-/-	-/-/-	-/-/-	-/-/-	-/-/-	-/-/-	-/-/-	n	n	n		
1c-RP3	MAP2v/3/4/5	ID	-/-/-	-/-/-	-/-/-	-/-/-	-/-/-	-/-/-	-/-/-	n	n	n		
1c-RQ5	MAP2v/3/4/5	ID	-/-/-	-/-/-	-/-/-	-/-/-	-/-/-	-/-/-	-/-/-	n	n	n		
1c-RL1	MAP2v/3/4/5	ID	-/-/-	-/-/-	+/2.0/+	+/4.0/+	+/4.5/+	+/4.0/+	+/2.0/+	3.5	2.5	1.5		
Average virus load of the unprotected/vaccinated cats at 24 wpc:									**3.0**	**4.5**	**4.0**	**2.0**		
2a-DV1	Adjuvant	SC/ID	-/-/-	-/-/+	+/2.5/+	+/3.0/+	+/3.0/+	+/4.0/+	+/4.5/+	6.0	4.0	6.0	1/3	6/16
2a-DV3	Adjuvant	SC/ID	-/-/-	-/-/-	+/2.5/-	+/3.0/+	+/3.0/+	+/3.5/+	+/4.0/+	5.0	6.0	2.5		
2a-OLN	Adjuvant	SC/ID	-/-/-	-/-/-	-/-/-	-/-/-	-/-/-	-/-/-	-/-/-	n	n	n		
2b-DV2	PBS	SC/ID	-/-/-	-/-/-	+/3.0/+	+/3.0/+	+/2.5/+	+/3.5/+	+/4.0/+	5.0	4.5	2.5	3/5	
2b-EA5	PBS	SC/ID	-/-/-	-/-/-	-/-/-	-/-/+	-/-/+	+/3.0/+	+/4.0/+	6.0	4.0	3.5		
2b-RO6	PBS	SC/ID	-/-/-	-/-/-	-/-/-	-/-/-	-/-/-	-/-/-	-/-/-	n	n	n		
2b-RP1	PBS	SC/ID	-/-/-	-/-/-	-/-/-	-/-/-	-/-/-	-/-/-	-/-/-	n	n	n		
2b-RQ4	PBS	SC/ID	-/-/-	-/-/-	-/-/-	-/-/-	-/-/-	-/-/-	-/-/-	n	n	n		
2c-RO3	Adjuvant	SC	-/-/-	-/-/-	-/-/-	-/-/-	-/-/-	-/-/-	-/-/-	n	n	n	1/3	
2c-RP2	Adjuvant	SC	-/-/-	-/-/-	-/-/+	+/2.5/+	+/4.0/+	+/5.0/+	+/4.5/+	6.0	5.5	5.0		
2c-RQ7	Adjuvant	SC	-/-/-	-/-/-	+/2.0/+	+/2.5/+	+/3.5/+	+/4.5/+	+/3.5/+	5.0	5.0	3.0		
2c-HOD	Adjuvant	ID	-/-/-	-/-/-	-/-/-	-/-/-	-/-/-	-/-/-	-/-/-	n	n	n	1/3	
2c-RQ2	Adjuvant	ID	-/-/-	-/-/-	+/2.0/+	+/3.0/+	+/4.0/+	+/4.5/+	+/5.0/+	4.5	4.5	3.5		
2c-RO5	Adjuvant	ID	-/-/-	-/-/-	+/1.0/+	+/2.5/+	+/2.0/+	+/2.5/+	+/2.0/+	4.5	1.5	0.5		
2d-DV5	n	n	-/-/-	-/-/-	-/3.0/+	-/2.5/+	-/3.0/+	-/3.0/+	-/3.5/+	5.0	5.0	2.5	0/2	
2d-HOI	n	n	-/-/-	-/-/-	+/3.0/+	+/1.0/+	+/2.5/+	+/1.5/+	+/0.5/+	3.0	1.5	0.5		
Average virus load of the infected control cats at 24 wpc:									**3.6**	**5.1**	**4.0**	**2.8**		

[a] Abbreviations: weeks post-challenge (wpc); bone marrow cells (BM); thymus cells (Th); mesenteric lymph node cells (mLN); Protection (Protect.); vaccine with MAP2v, MAP3, MAP4, and MAP5 (MAP2v/3/4/5); vaccine with MAP2, MAP2v, MAP3, and MAP5 (MAP2/2v/3/5); vaccine with MAP2, MAP3, and MAP4t (MAP2/3/4t) where MAP4t with Tat instead of palmitic acid; subcutaneous (SC); intradermal (ID); concurrent SC plus ID (SC/ID); negative (-); positive (+); not done (n).

[b] Proviral and virus load in PBMC; virus load number of X represents $10^X/10^6$ cells infected, with highest load of X=6 and undetectable load of X=0.

[c] Group with sub-group identification code is group number followed by sub-group alphabet code; sub-group shown with cat identification code.

[d] Sub-group 1a or Group 1a vaccination of (MAP2/±2v/3/±4/±5) means 1st vaccination with MAP2/3/4t and 2nd and 3rd vaccinations with MAP2/2v/3/5.

[e] Sub-groups 1b plus 1c or Group 1bc with all three vaccinations with MAP2v/3/4/5.

[f] Adjuvant supplemented with feline IL-12 for ID and concurrent SC/ID vaccinations.

Table S4. Immunogenicity of HIV-1 peptides counterpart to protective FIV peptides and FRT11 peptides

**HIV or FIV Pool & Peptide** [f]	**Counterpart HIV/FIV Pool & Peptide** [f]	**Responder Rate of HIV$^+$ Human Subjects (%) [Response Frequency]** [a]					**In Silico HLA Analyses (SB/SS) [WB/MS]** [b]		
		**Proliferation**		**Perf/GrzA/GrzB** [b]		**Cytokine**	**NetMHC 4.0** [c]	**NetCTL 1.2** [d]	**NetMHCII 2.3** [e]
		**CD4 T** [g]	**CD8 T** [g]	**CD4 CTL** [g]	**CD8 CTL** [g]	**IFNγ** [g]	**HLA-ABC** [bh]	**HLA-AB** [bhi]	**HLA-DRB1** [hj]
Hp10	Fp9	4/35 (11)	8/26 (31)	3/4 (75) [0.5000]	4/4 (100) [0.5833]	18/32 (58)	n	n	n
Hp10-1	Fp9-1	3/9 (33)	6/9 (67)	7/10 (70) [0.4667]	7/10 (70) [0.4333]	4/8 (50)	A2,B44,C15,A3C4,C5 A1,C12,A24,C14,C3	A24 [A2]	0401,1302,0802
Hp10-2	Fp9-2/**3**	1/9 (11)	**7/9 (78)**	**8/10 (80)** [**0.7000**]	**8/10 (80)** [**0.5333**]	2/8 (25)	A2,C15,A3,C5,A1 C12,B7,B83,C3	-	1302
**Hp10-3**	**Fp9-3**	0/9 (0)	6/9 (67)	5/10 (50) [0.4333]	6/10 (60) [0.4667]	**6/8 (75)**	B44,B7,B83	B44	[0103]
Hp15	Fp14	2/35 (6)	7/26 (27)	5/5 (100) [0.6667]	4/5 (80) [0.5556]	14/32 (44)	n	n	n
Hp15-1	Fp14-1	1/9 (11)	3/9 (33)	**9/10 (90)** [**0.3947**]	6/10 (60) [0.2632]	**5/8 (62)**	C15,C3,B58	B58	[-]
Hp15-2	Fp14-2	3/9 (33)	3/9 (33)	7/10 (70) [0.2632]	6/10 (60) [0.1579]	1/8 (12)	B27,A1,A24	A24,A26	[1501,0701, 1602,1001,0901]
**Hp15-3/4** [k]	**Fp14-3/4** [k]	2/9 (22)	**4/9 (44)**	8/10 (80) [0.2895]	**7/10 (70)** [**0.4211**]	4/8 (50)	B27,A1,A2 A24,B44	A24	[0802,0701,1602 1001,0401,1501, 1001,0901,1602]
HRT3	FRT3	6/26 (23)	9/24 (37)	4/6 (67) [0.6667]	5/6 (83) [0.5556]	5/32 (16)	n	n	n
HRT3-1	FRT3-1	**3/9 (33)**	3/9 (33)	9/13 (69) [0.5641]	9/13 (69) [0.5385]	1/17 (6)	B7,C7,A3,B27	A24	[1101,0801]
HRT3-2	FRT3-2	2/9 (22)	**4/9 (44)**	9/13 (69) [0.4872]	9/13 (69) [0.4872]	2/17 (12)	C3	[A3]	1101
**HRT3-3**	**FRT3-3**	2/9 (22)	**4/9 (44)**	9/13 (69) [0.4359]	**11/13 (85) [0.5897]**	**3/17 (18)**	A3,B27	[B27]	[-]
**HRT3-4**	**FRT3-4**	2/9 (22)	**4/9 (44)**	**11/13 (85) [0.6667]**	9/13 (69) [0.4615]	2/17 (12)	A3,B27	A3 [B27]	0405,1602
HRT3-5	FRT3-5	2/9 (22)	**4/9 (44)**	9/13 (69) [0.3846]	10/13 (77) [0.5641]	1/17 (6)	C7,B8,C6	B8,B62 [A26]	1101,0801
HRT7	FRT7	2/26 (12)	3/24 (12)	n	n	13/32 (41)	n	n	n
HRT11	FRT11	2/26 (8)	4/24 (17)	4/4 (100) [0.9167]	4/4 (100) [0.8333]	17/32 (53)	n	n	n
HRT11-1	FRT11-3	1/3 (33)	1/3 (33)	8/11 (73) [0.5758]	9/11 (82) [0.5758]	**3/3 (100)**	A2,A1	B58,A2,A26	0802
HRT11-2	FRT11-4	1/3 (33)	1/3 (33)	10/11 (91) [0.6364]	11/11 (100)[0.6061]	2/3 (67)	A1,A2,B62	A3,B62,B58 A1,A2	0802,0901,1302
HRT11-3	FRT11-5	1/3 (33)	1/3 (33)	7/11 (64) [0.4848]	9/11 (82) [0.4848]	2/3 (67)	A2,C3,C5	A3,B62,A2 [B44]	1602,0301,1501
**HRT11-4** [l]	FRT11-5/12-1 [l]	0/3 (0)	**2/3 (67)**	**11/11 (100) [0.7576]**	**11/11 (100) [0.6970]**	1/3 (33)	B8,C3,C6,A1/A3,A2	A3,B8,B39 [B62,A2]	1301,0103,1602 0301,1101,0701 1001,1501
HRT11-5 [m]	FRT12-1 [m]	1/3 (33)	1/3 (33)	4/4 (100) [0.5833]	3/4 (75) [0.5833]	1/3 (33)	A2,A1	B58,A2,A26	0802
FRT11	HRT10/11	3/26 (12)	6/24 (25)	4/4 (100) [0.7500]	3/4 (75) [0.5000]	5/32 (16)	n	n	n
FRT11-1	HRT10-4	2/3 (67)	**1/3 (33)**	10/11 (91) [0.7273]	**11/11 (100) [0.6970]**	ND	B44,C4,B8,A3,B27	B44 [B39]	[0301]
FRT11-2	HRT10-5	1/3 (33)	**1/3 (33)**	10/11 (91) **[0.7576]**	10/11 (91) [0.6061]	ND	B44,C4,A3,B27	B44 [B39]	[0301]
FRT11-3	HRT11-1	0/3 (0)	**1/3 (33)**	8/11 (73) [0.4545]	9/11 (82) [0.4848]	ND	A2	[A2]	0802,1101
FRT11-4	HRT11-2	1/3 (33)	0/3 (0)	9/11 (82) [0.6667]	9/11 (82) [0.5455]	ND	A1,C15,A2,A24/A1	A2,B58	0802
FRT11-5 [n]	HRT11-3/4 [n]	0/3 (0)	0/3 (0)	8/11 (73) [0.5152]	9/11 (82) [0.5152]	ND	A1,A3	B62,B58,A3	[-]

[a] Response frequency equals the average of the total positive responses divided by the average of the total responses.

[b] Abbreviations: Strong binder (SB); strong specificity or score (SS); weak binder (WB); moderate specificity or score (MS); $CD4^+$ (CD4 T) and $CD8^+$ T cell (CD8 T); $CD4^+$ CTL (CD4 CTL); $CD8^+$ CTL (CD8 CTL); granzyme A (GrzA), granzyme B (GrzB); HLA-A and HLA-B (HLA-AB) plus HLA-C (HLA-ABC), not done (n).

[c] HLA-A and HLA-B in single supertype or mixed supertypes (A1/A3,A24/A1), and HLA-C as C in two-digit resolution code using Net-MHC 4.0 server [20].

[d] CTL epitope prediction of HLA-A and HLA-B supertypes using NetCTL 1.2 server [24].

[e] Four-digit resolution nomenclature for HLA-DRB1 (DRB1) using Net-MHCII 2.3 server [22].

[f] RT peptide pool for HIV (HRT#) and FIV (FRT#); p24 peptide pool for HIV (Hp#) and FIV (Fp#); individual peptide shown as pool number-overlapping peptide number (Hp#-# or HRT#-#); bold HIV peptides as potential HIV vaccine immunogen; bold FIV peptides used in the current study.

[g] Individual peptide results are generally higher due to the use of higher numbers of HIV^+ subjects who are responders to the peptide pool.

[h] In order from the strongest to the weakest binding affinity (0.500-2.000 rank in NetMHC; 2.00-10.00 threshold in NetMHCII) or CTL score (>0.75 to >0.70 score or 0.970-0.964 specificity in NetCTL).

[i] Moderate specificity in bracket [supertype] shown with strong binder or alone when no strong binder available; no strong or weak binders (-).

[j] Weak binder in bracket [4-digit DRB1] shown when strong binder not present; no strong or weak binders [-].

[k] Hp15-3/4 (15mer) or overlapping peptide Hp15-3/Hp15-4 counterpart of an overlapping peptide Fp14-3/Fp14-4 (Fp14-3/4) (16mer).

[l] HRT11-4 (15mer) counterpart of FRT11-5 (15mer) based on 10 aa on the amino-end but with 10 aa overlap on the carboxyl-end with FRT12-1.

[m] HRT11-5 (15mer) counterpart of FRT12-1 (15mer) with 10 aa overlap on the amino-end with FRT11-5.

[n] HRT11-3/4 (14mer) represents HRT11-3 (14mer) with 10 aa overlap on the carboxyl-end with HRT11-4 and serves as counterpart of FRT11-5.

Table S5. Responder rate and average magnitude of the MAP/peptide responses by the unprotected cats from all vaccine groups

**MAP & Peptide**	**3-6w Post-2nd Vac [a]**		**3w Post-3rd Vac [a]**		**6w Post-3rd Vac [a]**		**3-6w Post-2nd Vac [a]**		**3w Post-3rd Vac [a]**		**6w Post-3rd Vac [a]**	
	**CD4 T [b]**	**CD8 T [b]**	**CD4 T [b]**	**CD8 T [b]**	**CD4 T [b]**	**$CD8^+$ T [b]**	**IL-2 [b]**	**IFNγ**	**IL-2 [b]**	**IFNγ**	**IL-2 [b]**	**IFNγ**
**Percent responder rate [c]**												
MAP4	50.0	0.0	0.0	**50.0**	100.0 [f]	0.0 [f]	0.0	0.0	0.0	50.0	0.0	50.0
Fp9-3	0.0	0.0	25.0	**75.0**	0.0 [f]	0.0 [f]	0.0	25.0	0.0	0.0	0.0	0.0
MAP3	0.0	0.0	25.0	**25.0**	25.0	**50.0**	50.0	100.0	25.0	100.0	0.0	75.0
Fp14-3	0.0	0.0	25.0	0.0	75.0	0.0	25.0	75.0	25.0	100.0	0.0	25.0
Fp14-4	0.0	**50.0**	0.0	0.0	25.0	**25.0**	75.0	100.0	50.0	100.0	0.0	50.0
MAP2v	50.0	25.0	75.0	50.0	0.0	**75.0**	**100.0**	75.0	0.0	100.0	**50.0**	50.0
FRT3-3	25.0	0.0	0.0	0.0	75.0	**75.0**	25.0	50.0	**50.0**	50.0	**50.0**	50.0
FRT3-4	0.0	**25.0**	25.0	**25.0**	100.0	75.0	0.0	75.0	50.0	100.0	25.0	50.0
MAP5	25.0	**25.0**	25.0	0.0	100.0	0.0	0.0	0.0	0.0	25.0	0.0	0.0
FRT7-1	25.0	0.0	0.0	**25.0**	0.0	**50.0**	0.0	0.0	0.0	25.0	0.0	0.0
FRT7-2	25.0	**25.0**	0.0	0.0	25.0	**25.0**	**25.0**	25.0	0.0	25.0	0.0	0.0
Average	18.2	13.6	18.2	22.7	47.2	41.1	27.3	47.7	18.2	61.4	11.4	31.8
p-value [e]		*0.5703*		*0.6673*		*0.7469*	*0.2099*		***0.0061***		*0.0636*	
**Average magnitude of positive responses [d]**												
MAP4	1.9	0.0	0.0	**111.5**	9.8 [f]	0.0 [f]	0.0	0.0	0.0	55.5	0.0	55.5
Fp9-3	0.0	0.0	4.0	**21.0**	0.0 [f]	0.0 [f]	0.0	314.0	0.0	0.0	0.0	0.0
MAP3	0.0	0.0	5.0	2.0	3.0	**5.0**	151.5	280.6	233.0	434.0	0.0	142.7
Fp14-3	0.0	0.0	12.5	0.0	2.6	0.0	**148.0**	119.0	99.0	183.3	0.0	200.0
Fp14-4	0.0	**44.1**	0.0	0.0	1.0	**2.0**	90.3	205.1	127.5	264.0	0.0	172.5
MAP2v	14.5	**19.0**	1.9	**11.4**	0.0	**14.0**	222.5	234.3	0.0	240.3	86.0	371.5
FRT3-3	0.0	0.0	0.0	0.0	4.4	2.5	87.0	512.5	53.3	733.5	76.3	333.0
FRT3-4	1.0	**7.1**	1.0	**1.9**	12.3	10.0	0.0	222.3	77.0	318.5	**218.0**	132.0
MAP5	7.4	**10.9**	3.9	0.0	2.4	0.0	0.0	0.0	0.0	181.0	0.0	0.0
FRT7-1	3.4	0.0	0.0	**14.5**	0.0	**15.7**	0.0	0.0	0.0	52.0	0.0	0.0
FRT7-2	2.4	2.1	0.0	0.0	3.0	1.0	77.0	108.0	0.0	50.0	0.0	0.0
Average	2.8	7.6	2.6	14.8	3.2	5.6	70.6	181.4	53.6	228.4	34.6	127.9
p-value [e]		*0.2900*		*0.2498*		*0.3344*	*0.0551*		***0.0245***		*0.0578*	

[a] At 3, 6, or 3-6 week (w) post-2nd or -3rd vaccination (Vac).
[b] Bold value when $CD8^+$ T-cell (CD8 T) proliferation or IL-2 production is ≥$CD4^+$ T-cell (CD4 T) proliferation or ≥IFNγ production, respectively.
[c] Percent responder rate: the number of responders to the peptide divided by the total number of responders multiplied by 100.
[d] Magnitude of $CD4^+$ T cell or $CD8^+$ T cell proliferation in average % $CFSE^{low}$; magnitude of IFNγ and IL-2 production in average SFU/10^6 PBMC.
[e] Statistics between $CD4^+$ and $CD8^+$ T-cell proliferation and between IL-2 and IFNγ production at each time-point with a significance at $p<0.05$ in bold.
[f] Excluded from the average due to small sample size of one unprotected/vaccinated cat.

Table S6. Responder rate and average magnitude of the MAP/peptide responses by the protected cats from vaccine Group 1bc

MAP &	3-6wk Post-2nd Vac[a]		3wk Post-3rd Vac[a]		6wk Post-3rd Vac[a]		3-6wk Post-2nd Vac[a]		3wk Post-3rd Vac[a]		6wk Post-3rd Vac[a]	
Peptide	CD4 T[b]	CD8 T[b]	CD4 T[b]	CD8 T[b]	CD4 T[b]	CD8 T[b]	IL-2[b]	IFNγ[b]	IL-2[b]	IFNγ[b]	IL-2[b]	IFNγ[b]
**Percent responder rate[c]**												
MAP4	38.5	**46.2**	53.8	**76.9**	84.6	**84.6**	**7.7**	7.7	15.4	23.1	**7.7**	0.0
Fp9-3	30.8	23.1	38.5	**76.9**	30.8	**53.8**	23.1	7.7	0.0	15.4	**7.7**	0.0
MAP3	46.2	15.4	69.2	46.2	38.5	**38.5**	**46.2**	30.8	**46.2**	30.8	**53.8**	46.2
Fp14-3	53.8	23.1	76.9	61.5	100.0	53.8	**46.2**	30.8	23.1	38.5	23.1	53.8
Fp14-4	23.1	7.7	61.5	46.2	46.2	**46.2**	**69.2**	61.5	30.8	38.5	15.4	38.5
MAP2v	38.5	**53.8**	69.2	53.8	84.6	61.5	**46.2**	30.8	**46.2**	38.5	15.4	30.8
FRT3-3	30.8	7.7	30.8	**53.8**	23.1	**53.8**	**69.2**	53.8	15.4	30.8	7.7	15.4
FRT3-4	23.1	**23.1**	76.9	53.8	92.3	61.5	0.0	15.4	**46.2**	30.8	**69.2**	69.2
MAP5	61.6	30.8	69.2	53.8	84.6	53.8	**30.8**	15.4	**7.7**	7.7	**23.1**	7.7
FRT7-1	69.2	46.2	84.6	46.2	92.3	69.2	46.2	53.8	15.4	23.1	0.0	0.0
FRT7-2	30.8	23.1	84.6	46.2	61.5	**76.9**	69.2	100	**23.1**	23.1	15.4	30.8
Average	40.6	27.3	65.7	55.9	67.1	59.4	**41.3**	38.5	24.5	27.3	21.7	26.6
p-value[e]		*0.0575*		*0.1309*		*0.4221*	*0.7092*		*0.6299*		*0.6163*	
**Average magnitude of positive response[d]**												
MAP4	12.6	7.2	7.4	**13.5**	9.7	**13.3**	**150**	63	60	76	**493**	0
Fp9-3	3.8	**10.9**	3.1	**7.9**	6.5	**8.5**	114	78	0	72	**199**	0
MAP3	3	**3.6**	6.2	**8.1**	7.0	6.8	**130**	113	178	438	170	237
Fp14-3	3.6	**4.6**	31.7	10.4	21.8	5.6	104	120	228	241	171	183
Fp14-4	2.3	1.9	5.9	4.2	5.9	2.7	186	248	70	123	127	176
MAP2v	8.5	**29.7**	9.7	**14.3**	11.4	9.5	170	362	240	780	119	356
FRT3-3	2.3	1.2	4.8	**9.1**	5.8	**6.4**	**111**	13	229	398	117	512
FRT3-4	4.4	**5.4**	8.7	4	9.4	6.5	0	70	**397**	243	**324**	272
MAP5	7.6	3.9	14.1	7.8	14.7	6.7	**102**	85	58	98	63	79
FRT7-1	12.4	8.2	14.1	**14.4**	10.4	9.8	93	96	**119**	61	0	0
FRT7-2	3.8	**6.5**	11.0	4.8	16.3	7.6	**216**	200	111	113	**142**	81
Average	5.8	**7.5**	10.6	9.0	10.8	7.6	125	142.3	153.6	240.3	175.0	172.3
p-value[e]		*0.5272*		*0.5400*		*0.0799*	*0.8532*		*0.2677*		*0.9674*	

[a] At 3, 6, or 3-6 week (w) post-2nd or 3rd vaccination (Vac).
[b] Bold value when $CD8^+$ T-cell (CD8 T) proliferation or IL-2 production is ≥$CD4^+$ T-cell (CD4 T) proliferation or ≥IFNγ production, respectively.
[c] Percent responder rate: the number of responders to the peptide divided by the total number of responders multiplied by 100.
[d] Magnitude of $CD4^+$ T cell or $CD8^+$ T cell proliferation in average % $CFSE^{low}$; magnitude of IFNγ and IL-2 production in average SFU/10^6 PBMC.
[e] Statistics between $CD4^+$ and $CD8^+$ T-cell proliferation and between IL-2 and IFNγ production at each time-point with a significance at $p<0.05$.

Table S7. Comparing the responder rates between the selection study and MAP vaccine trial and between the post-2nd and post-3rd vaccinations

	Selection Study		MAP Vaccine Trial					
	3-4wk Post-4th Vac [a]		3wk Post-3rd Vac [a]		6wk Post-3rd Vac [a]		3-6wk Post-2nd Vac [a]	
**FIV Peptide**	**CD4 T [b]**	**CD8 T [b]**	**CD4 T [b]**	**CD8 T [b]**	**CD4 T [b]**	**CD8 T [b]**	**CD4 T [b]**	**CD8 T [b]**
**Percent Responder Rate [c]**								
Fp9-3	66.7	**66.7**	38.5	**76.9**	30.8	**53.8**	30.8	23.1
Fp14-3	33.3	**33.3**	76.9	61.5	100.0	53.8	53.8	23.1
Fp14-4	33.3	**33.3**	61.5	46.2	46.2	**46.2**	23.1	7.7
FRT3-3	42.9	**42.9**	30.8	**53.8**	23.1	**53.8**	30.8	7.7
FRT3-4	42.9	**57.1**	76.9	53.8	92.3	61.5	23.1	**23.1**
FRT7-1	100.0	**100.0**	84.6	46.2	92.3	69.2	69.2	46.2
FRT7-2	66.7	**66.7**	84.6	46.2	61.5	**76.9**	30.8	23.1
Average:	55.1	57.1	64.8	54.9	63.7	59.3	37.4	22.0
p-value [d] (CD4 T *vs.* CD8 T):	-	*0.8768*	-	*0.3195*	-	*0.7352*	-	*0.0872*
[e] (Select Study *vs.* Vac Trial):	-	-	*0.4491*	*0.8293*	*0.1446*	*0.8300*	*0.5780*	***0.0070***
[f] (Post-2nd *vs.* 3wk Post-3rd):	-	-	-	-	-	-	***0.0250***	***0.0003***
[g] (Post-2nd *vs.* 6wk Post-3rd):	-	-	-	-	-	-	*0.0841*	***<0.0001***

[a] At 3, 6, or 3-6 week (wk) post-2nd or 3rd vaccination (Vac).
[b] Bold value when $CD8^+$ T-cell (CD8 T) proliferation is greater than or equal to $CD4^+$ T cell (CD4 T) proliferation.
[c] Percent responder rate: the number of responders to the peptide divided by the total number of responders multiplied by 100.
[d] Statistics between the $CD4^+$ and $CD8^+$ T-cell proliferation at each time-point with a significance at $p<0.05$.
[e] Statistical analysis of the $CD4^+$ and $CD8^+$ T-cell proliferation values between the Selection Study and the MAP Vaccine Trial.
[f] Statistics between the 3-6 weeks post-2nd and 3 weeks post-3rd vaccinations with a significance at $p<0.05$ in bold italics.
[g] Statistics between the 3-6 weeks post-2nd and 6 weeks post-3rd vaccinations with a significance at $p<0.05$ in bold italics.

Table S8. Primers and probes for qRT-PCR

Primers and Probes [a]	Sequence
Primers [b]	
GAPDH_for	ATGTTCCAGTATGATTCCACCC
GAPDH_rev	ACCAGCATCACCCCATTTG
GrzA_for	CCTATACATTTCTGGCATCCTCTC
GrzA_rev	CGGCACAGATACTTTCTCCTT
GrzB_for	AAGAGAAGTTCGTGCTGACAG
GrzB_rev	TTCTTTGGATTATAGTCTGGGTGG
IFNγ_for	AATACCAGCTCCAGTAAACGG
IFNγ_rev	GCTTCCTCAGGTTAGATCTTGG
IL2_for	CTACAAAGGAAACACAGCAACAG
IL2_rev	TGAGCATCCTGGAGAGTTTG
CD107a_for	CCTTCGTGACCAGCTATGATAC
CD107a_rev	GTCCTCTTCCAAAAGCAATCAC
Perforin_for	ACAACCCATGCCAGTGTG
Perforin_rev	TCCTAGTGAAAGAGTCTCCCC
Probes [c]	
GAPDH	AAATTCCACGGCACAGTCAAGGC
GrzA	AGCACCATGTAGGGTCTTGAATGAGG
GrzB	TCTCCTGCTTCTTGATGTTGTGGGC
IFNγ	CAGGTCCAGCGCAAAGCAATAAATGA
IL2	CCATTCAAAAGCAACCGTAAATCCAGCA
CD107	TTCGACCTACCACCTGATGCAGC
Perforin	TCATGAACTTCCAGGCGTCAGGC

[a] Abbreviations: glyceraldehyde 3-phosphate dehydrogenase (GAPDH), granzyme A (GrzA), granzyme B (GrzB), interferon-γ (IFNγ), interleukin-2 (IL-2).

[b] Forward primer (_*for*); reverse primer (_*rev*).

[c] All probes are dual labeled with FAM fluorescein and blackhole quencher 1.
